# Breastfeeding Affects Concentration of Faecal Short Chain Fatty Acids During the First Year of Life: Results of the Systematic Review and Meta-Analysis

**DOI:** 10.3389/fnut.2022.939194

**Published:** 2022-07-11

**Authors:** Igor Łoniewski, Karolina Skonieczna-Żydecka, Laura Stachowska, Magdalena Fraszczyk-Tousty, Piotr Tousty, Beata Łoniewska

**Affiliations:** ^1^Department of Biochemical Science, Pomeranian Medical University in Szczecin, Szczecin, Poland; ^2^Department of Human Nutrition and Metabolomics, Pomeranian Medical University in Szczecin, Szczecin, Poland; ^3^Department of Neonatal Diseases, Pomeranian Medical University in Szczecin, Szczecin, Poland; ^4^Department of Obstetrics and Gynecology, Pomeranian Medical University in Szczecin, Szczecin, Poland

**Keywords:** microbiota, metabolome, gut, infant, feeding

## Abstract

Short chain fatty acids (SCFAs) are important metabolites of the gut microbiota. It has been shown that the microbiota and its metabolic activity in children are highly influenced by the type of diet and age. Our aim was to analyse the concentration of fecal SCFAs over two years of life and to evaluate the influence of feeding method on the content of these compounds in feces. We searched PubMed/MEDLINE/Embase/Ebsco/Cinahl/Web of Science from the database inception to 02/23/2021 without language restriction for observational studies that included an analysis of the concentration of fecal SCFAs in healthy children up to 3 years of age. The primary outcome measures-mean concentrations-were calculated. We performed a random-effects meta-analysis of outcomes for which ≥2 studies provided data. A subgroup analysis was related to the type of feeding (breast milk vs. formula vs. mixed feeding) and the time of analysis (time after birth). The initial search yielded 536 hits. We reviewed 79 full-text articles and finally included 41 studies (*n* = 2,457 SCFA analyses) in the meta-analysis. We found that concentrations of propionate and butyrate differed significantly in breastfed infants with respect to time after birth. In infants artificially fed up to 1 month of age, the concentration of propionic acid, butyric acid, and all other SCFAs is higher, and acetic acid is lower. At 1–3 months of age, a higher concentration of only propionic acid was observed. At the age of 3–6 months, artificial feeding leads to a higher concentration of butyric acid and the sum of SCFAs. We concluded that the type of feeding influences the content of SCFAs in feces in the first months of life. However, there is a need for long-term evaluation of the impact of the observed differences on health later in life and for standardization of analytical methods and procedures for the study of SCFAs in young children. These data will be of great help to other researchers in analyzing the relationships between fecal SCFAs and various physiologic and pathologic conditions in early life and possibly their impact on health in adulthood.

## Introduction

During the first 2–3 years of life, the gut microbiome undergoes rapid and important changes in bacterial community structure and function (1, 2). This maturation phase is characterized by an early abundance of *Bifidobacterium, Bacteroides*, and *Escherichia*, which are gradually replaced by obligate anaerobic bacteria, particularly members of the Firmicutes phylum, such as *Clostridiaceae* and *Lachnospiraceae* (3). One of the most important factors in the composition of the gut of infants is human milk in the diet of infants (1, 2). Consumption of breast milk is associated with increased abundance of *Bifidobacteria* and decreased abundance of Firmicute *Lachnospiracea* (4, 5). Formula-fed infants tend to be richer in *Bacteroides, Escherichia, Enterobacteriaceae, Clostridium* (1, 6) and other bacteria associated with a more mature microbiota (7). Some evidence exists that bacterial colonization of the gut by breast milk bacteria is dose-dependent. Pannaraj et al. found that infants whose diet consisted of 75% human milk (>) obtained about 27% of their gut bacteria from breast milk, whereas infants who were less breastfed obtained only about 17% of their bacteria from milk-this difference in bacterial uptake decreases as infants get older and are exposed to other bacterial sources (8). Infant gut colonization is also influenced by other factors, including cesarean delivery (9) antibiotic use (10) and maternal body mass index (BMI) (11). Like formula feeding, these factors are associated with early intestinal maturation (i.e., higher numbers of Firmicutes), which may have adverse effects on immune system development (12, 13) and may increase the risk of obesity in children (11, 14).

Microorganisms are linked to diet and various physiological situations through their ability to produce gut microbial metabolites. Since humans lack the enzymes to break down indigestible carbohydrates, these carbohydrates are fermented by bacterial species in the gastrointestinal tract in the cecum and colon (15). This phenomenon produces various metabolites such as short-chain fatty acids (SCFAs) as the main group (16). In bacteria themselves, SCFAs are a waste product essential for the formation of the redox equivalent in the anaerobic situation (17). These metabolites are organic acids with one to six carbon atoms, including acetate (C2), propionate (C3), and butyrate (C4) (18). SCFAs have various and crucial activities in humans; in this regard, butyrate is probably the best known SCFA for health and disease (19). Butyrate is the most important energy pool for colonocytes. This metabolite plays an influential role in cancer control by stimulating cell apoptosis and modulating gene expression *via* histone deacetylases (HDACs) (20) In addition, propionate is also an energy pool for many cells, such as epithelial cells, and could be transferred to the liver where it is involved in gluconeogenesis (19). Lastly, another SCFA, acetate, could enter peripheral tissues and be involved in lipogenesis and cholesterol metabolism. Recent *in vivo* documents show that it also plays a crucial role in central appetite modulation (21). On the other hand, SCFAs have been shown to influence bacterial gene expression. In this context, acetate induces gene expression in *Salmonella typhimurium*, for example, which occurs through bacterial invasion due to the formation of acetyl phosphate (22). In addition, exposure of enterohaemorrhagic *Escherichia coli* (EHEC) to butyrate has been found to induce gene expression involved in adherence to epithelial cells *via* recognition of Lrp, the leucine-responsive regulatory protein (23).

Creating gut microbiota occurs primarily in the period of infancy. *Bifidobacterium* dominates during breastfeeding whilst in case of formula feeding *Bacteroides* and *Clostridioides* abundance is higher. A milestone in creating the microbiome is the introduction of solid foods, usually around 6 months of age. Consistently, over time and depending on the infant's diet, there are differences in the concentration of short-chain fatty acids (SCFAs)—the main metabolites of the microbiota. It has been shown that infants who were exclusively breastfed had lower levels of total SCFA: propionate, butyrate, iso butyrate, valerate and isovalerate, and higher levels of intermediate metabolites such as lactate and succinate. In addition, a higher relative acetic acid content was observed. This information is based on single observational studies and no systematic review with meta-analysis has been performed so far. Therefore, we decided to perform a systematic review and meta-analysis of the concentration of SCFAs (propionic, butyric, acetic) in the first 3 years of life and analyse the changes in these values over time and regarding the diet type (breastfeeding vs. formula). These data will be of great help to other researchers in analyzing the relationships of fecal SCFAs with various physiological and pathological conditions at early in life and potentially their effects on health in adulthood. They will also be helpful in comparing the effects of dietary interventions, new formula composition, the use of pre- and probiotics, and drugs on the fecal concentration of SCFAs.

## Methods

### Search Strategy and Inclusion Criteria

We commissioned three independent authors (LS, MFT, PT) to search PubMed/MEDLINE/Embase/Ebsco/Cinahl/Web of Science from database inception to 23/02/2021 without language restriction for observational studies involving analysis of SCFA levels in healthy children up to 3 years of age. We also included randomized controlled trials (RCTs) that compared a specific intervention with placebo/no intervention, the latter group being of interest.

The following search terms were used.

*String for Pub Med./Cinahl/Web Of Science:* (newborn OR child, newborn OR full term infant OR human neonate OR human newborn OR infant, newborn OR neonate OR neonatus OR newborn OR newborn baby OR newborn child OR newborn infant OR newly born baby OR newly born child OR newly born infant OR child OR child OR children) AND (feces OR fecal excretion OR feces OR fecal excretion OR feces OR stool OR stools) AND (short chain fatty acid OR fatty acid, short chain OR short chain fatty acid OR acetic acid OR acetate OR acetate salt OR acetate sodium OR acetic acid OR sodium acetate OR butyric acid OR butanoate sodium OR butanoic acid OR butyrate OR butyrate sodium OR butyric acid OR n butyrate OR n butyric acid OR sodium butyrate OR sodium n butyrate OR propionic acid OR propionate OR propionic acid) AND (observational study OR non experimental studies OR non experimental study OR nonexperimental studies OR nonexperimental study OR observation studies OR observation study OR observational studies OR observational study OR observational study as topic OR observational studies as topic OR cross-sectional study OR cross-sectional design OR cross-sectional research OR cross-sectional studies OR cross-sectional study OR cohort analysis OR analysis, cohort OR cohort analysis OR cohort life cycle OR cohort studies OR cohort study OR randomized controlled trial OR controlled trial, randomized OR randomized controlled study OR randomized controlled trial OR randomized controlled study OR randomized controlled trial OR trial, randomized controlled).

In PubMed: filter: *HUMANS*

*String for Embase:* (‘newborn'/exp OR ‘child, newborn' OR ‘full term infant' OR ‘human neonate' OR ‘human newborn' OR ‘infant, newborn' OR ‘neonate' OR ‘neonatus' OR ‘newborn' OR ‘newborn baby' OR ‘newborn child' OR ‘newborn infant' OR ‘newly born baby' OR ‘newly born child' OR ‘newly born infant' OR ‘child'/exp OR ‘child' OR ‘children') AND (‘feces'/exp OR ‘fecal excretion' OR ‘faeces' OR ‘fecal excretion' OR ‘feces' OR ‘stool' OR ‘stools') AND (‘short chain fatty acid'/exp OR ‘fatty acid, short chain' OR ‘short chain fatty acid' OR ‘acetic acid'/exp OR ‘acetate' OR ‘acetate salt' OR ‘acetate sodium' OR ‘acetic acid' OR ‘sodium acetate' OR ‘butyric acid'/exp OR ‘butanoate sodium' OR ‘butanoic acid' OR ‘butyrate' OR ‘butyrate sodium' OR ‘butyric acid' OR ‘n butyrate' OR ‘n butyric acid' OR ‘sodium butyrate' OR ‘sodium n butyrate' OR ‘propionic acid'/exp OR ‘propionate' OR ‘propionic acid') AND (‘observational study'/exp OR ‘non experimental studies' OR ‘non experimental study' OR ‘nonexperimental studies' OR ‘nonexperimental study' OR ‘observation studies' OR ‘observation study' OR ‘observational studies' OR ‘observational study' OR ‘observational study as topic' OR ‘observational studies as topic' OR ‘cross-sectional study'/exp OR ‘cross-sectional design' OR ‘cross-sectional research' OR ‘cross-sectional studies' OR ‘cross-sectional study' OR ‘cohort analysis'/exp OR ‘analysis, cohort' OR ‘cohort analysis' OR ‘cohort life cycle' OR ‘cohort studies' OR ‘cohort study' OR ‘randomized controlled trial'/exp OR ‘controlled trial, randomized' OR ‘randomized controlled study' OR ‘randomized controlled trial' OR ‘randomized controlled study' OR ‘randomized controlled trial' OR ‘trial, randomized controlled').

We supplemented the electronic search with a manual review of reference lists of relevant meta-analyses and reviews.

The inclusion criteria were:

- data on the concentration of SCFAs in stool,
- age 0–3 years.

We excluded reviews, systematic reviews, editorials, single case reports, opinion articles and editorials/perspectives.

### Data Abstraction

The protocol for this systematic review, meta-analysis and meta-regression was registered in the International Prospective Register of Systematic Reviews (Prospero database registration number CRD42022313244). Data on the study design and study group of each study were extracted by two independent reviewers (MFT, LS) in accordance with the PRISMA (Preferred Reporting Items for Systematic Reviews and Meta-Analyses) standard. If data were missing for the review, the authors were contacted twice by email at 2-week intervals to obtain additional information. Disagreements were resolved by consensus, involving a clinical lead (BŁ).

### Outcomes

Co-primary endpoints were the concentrations of: (i) acetic acid, (ii) propionic acid, (iii) butyric acid and (iv) all SCFAs. Secondary outcomes were concentrations of other SCFAs, like valeric, isobutyric, caproic, and others.

### Data Synthesis and Statistical Analysis

We conducted a random effects (24) meta-analysis of outcomes for which ≥3 studies contributed data, using Comprehensive Meta-Analysis V3 (http://www.meta-analysis.com). We explored study heterogeneity using the chi-square test of homogeneity, with *p* < 0.05 indicating significant heterogeneity. All analyses were two-tailed with alpha = 0.05.

Group differences in continuous outcomes were analyzed as the pooled means. A subgroup analyses regarding feeding type (breast vs. formula vs. mixed) and time of analysis (time post delivery) were conducted. We utilized the following time ranges: up to ≤ 1 month, >1– ≤ 3 months, >3– ≤ 6 months, >6– ≤ 9 months, >9– ≤ 12 months and >12 months of age. Categorical outcomes were not analyzed. Finally, we inspected funnel plots and used Egger's regression test (25) and the Duval and Tweedie's trim and fill method (26) to quantify whether publication bias could have influenced the results.

### Risk of Bias

As our systematic review and meta-analysis contains single arms form RCTs predominantly (involving healthy children only, receiving placebos and also data abstracted from control groups (healthy children) in case control-studies, we decided not to evaluate the bias, as such score refers to a whole study, not its part.

## Results

### Search Results

**Figure 1:**
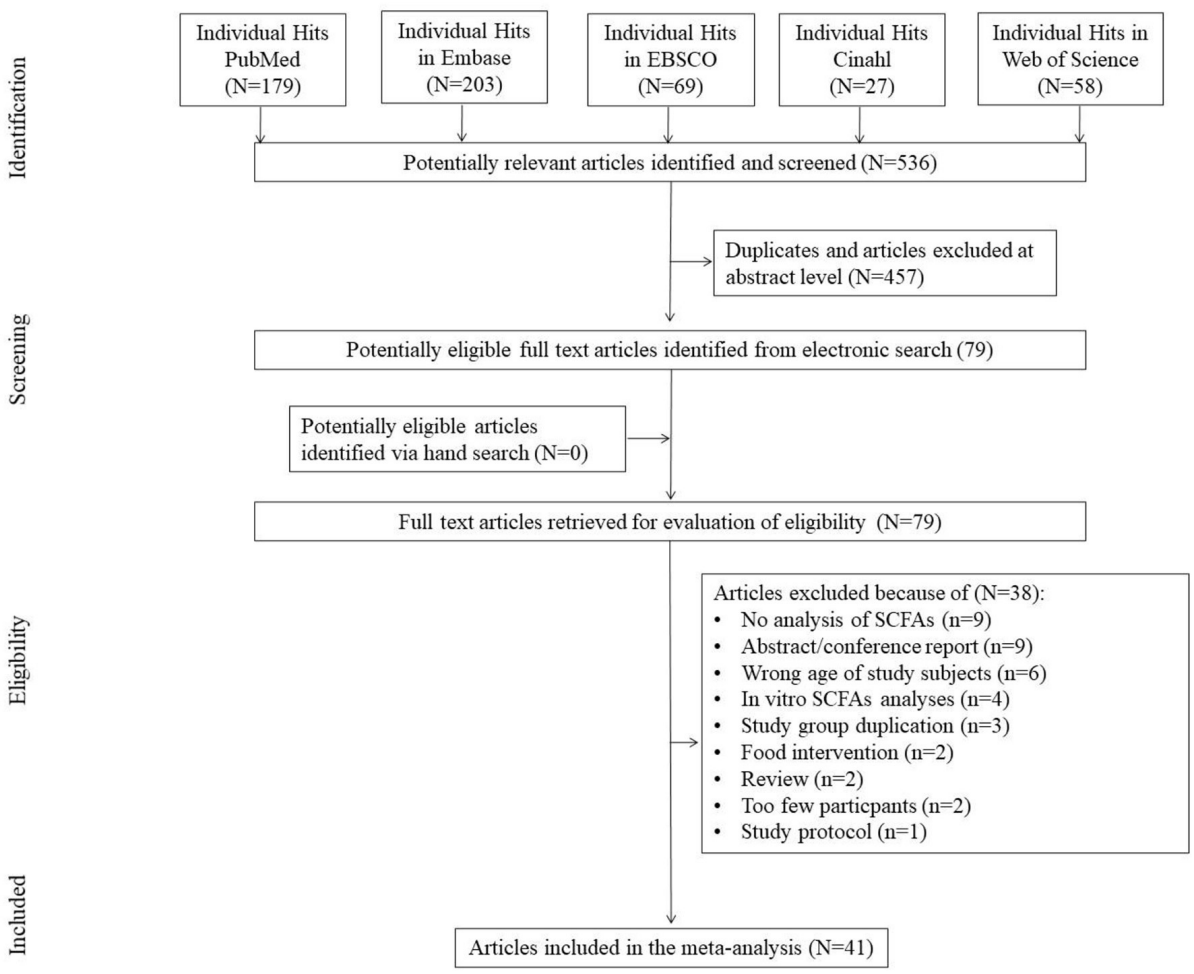
Study flow chart.

The first search yielded 536 hits. Four hundred fifty-seven studies were excluded because they were duplicates and/or because they were assessed at the title/abstract level. No additional articles were identified *via* the hand search. Two studies that qualified for meta-analysis included data from sources indicated by the authors of the publication (27, 28). Subsequently, all 79 full-text articles were reviewed. Of these, 38 were excluded because they did not meet the inclusion criteria. The reasons for exclusion were: no analysis of SCFAs/no data available (*N* = 9), abstract/conference report (*N* = 9), wrong age of subjects studied (*N* = 6), *in vitro* analyses (*N* = 4), duplicate study group (*N* = 3), food intervention (*N* = 2), review (*N* = 2), too few participants (*N* = 2), study protocol (*N* = 1) (Figure 1), leaving 41 studies included in the meta-analysis.

### Characteristics of the Studies and the People Studied

A total of 41 studies were included with a total number of *n* = 2,457 SCFAs analyses. We pooled data on controls from case-control (29–35), cohort (36–47) and cross-sectional studies (48–51). Data on children from RCTs control groups were also included (52–69). There were both term and pre-term delivered children (but only healthy) included. Other data, delivery type and nutrition method are placed in Table 1. Please note that the subgroups in the first column reflect the data provided by the authors according to the time of the analyses, which correspond to the data in the forest plots.

**Table 1:** Study groups characteristics.

**ID**	**References/** **type of study**	**Aim**	**Specification of healthy controls**	***N* of subjects/** **N whole study group/** **visits in case of multiple analyses**.	**T/PT: HBD** **(mean ±SD), weeks**.	**Delivery type *N* (cesearan/** **vaginal)**	**Nutrition method in infancy**
1	Ben et al. (29)/case control	Effect of GOS on intestinal microbiota.	Receiving formula	24/164. Visit at age: 3 m.	T	ND	MF:45
1a			Receiving human milk.	17/164. Visit at age: 3 m.	T		BF:24
2	Ben et al. (30)/case control	The effect of infant formula milk consisting of GOS on intestinal microbiota and the fermentation characteristics in term infants in comparison with that of human milk	Receiving breastfeeding.	26/271. Visit at age: 3 m, 6 m.	ND	ND	BF:26
2a							
2b			Receiving formula.	52/271. Visit at age: 3 m, 6 m.			MF:52
2c							
3	Berni Canani et al. (48)/cross-sectional	To evaluate gut microbiota composition and butyrate production in children affected by non-IgE-mediated cow's milk allergy.	Healthy children at the mean age 12.9 ± 7.4 months	23/69	ND	10/13	BF (for at least 1 mth):14
4	Brink et al. (36)/cohort	To investigate fecal microbiota and metabolites at different ages in infants who were breastfed (BF), received cow milk—based formula (MF), or received soy-based formula (SF) or no longer breastfeeding (NLB).	Receiving breastfeeding	Visit at age 3 m: 16/42	T	ND	BF:16
4a				Visit at age 6 m: 20/54	T		BF:20
4b				Visit at age 9 m: 12/35	T		BF:35
4c				Visit at age 12 m:14/53	T		BF:14
4d			Receiving milk formula	Visit at age 3 m: 12/42	T		MF:12
4e				Visit at age 6 m: 19/54	T		MF:19
4f				Visit at age 9 m: 11/35	T		MF:11
4g				Visit at age 12 m: 14/53	T		MF:!4
4h			Receiving soya based formula	Visit at age 3 m: 14/42	T		MF:14
4i				Visit at age 6 m: 15/54	T		MF:15
4j				Visit at age 9 m: 12/35	T		MF:12
4k				Visit at age 12 m: 15/53	T		MF:15
4l			Switched to milk formula when breastfeeding was not possible.	Visit at age 12 m:10/53	T		MF:10
5	Bazanella et al. (52)/RCT	To determine the effects of a bifidobacteria-containing formula on the healthy human intestinal microbiome during the first year of life	Receiving placebo formula	49/106 Visits at age: 1, 3, 5, 7, 9, 12 month.	T	27/22	MF:11; BF:38.
5a							
5b							
5c			Receiving breastfeeding	9/106 Visits at age: 1, 3, 5, 7, 9, 12 month.	T	6/3	BF:9.
5d							
5e							
5a							
5b							
5c							
5d							
5e							
6	Bakker-Zierikzee et al. (31)/case control	To compare the effects of infant formula containing a mixture of galacto- and fructo-oligosaccharides or viable *Bifidobacterium animalis* on the composition and metabolic activity of the intestinal microbiota.	Receiving breast milk	Visit at age 5 days: lactate 43/120, SCFA 32/120. Visit at age 10 days: lactate 44/120, SCFA 33/120. Visit at age 4 weeks: lactate 35/120, SCFA 28/120. Visit at age 8 weeks: lactate 31/120, SCFA 22/120. Visit at age 12 weeks: lactate 26/120, SCFA 24/120. Visit at age 16 weeks: lactate 22/120, SCFA 17/120.	ND	Total: 4/59	BM:63
6a							
6b							
6c							
6d							
6e							
6f			Receiving standard formula.	Visit at age 5 days: lactate 13/120, SCFA 12/120. Visit at age 10 days: lactate 19/120, SCFA 15/120. Visit at age 4 weeks: lactate 16/120, SCFA 13/120. Visit at age 8 weeks: lactate 15/120, SCFA 14/120. Visit at age 12 weeks: lactate 15/120, SCFA 14/120. Visit at age 16 weeks: lactate 14/120, SCFA 12/120.		Total: 5/14	Total: MF:19
6g							
6h							
6i							
6j							
6k							
7	Díaz et al. (37)/cohort	To establish potential links between type of formula substitutes, microbiota and desensitization in infants with Non-IgE mediated cow's milk protein allergy.	10 age-matched healthy infants (12–24 months old), with a normal diet consuming cow's milk	10/27	ND	ND	MF
8	Differding et al. (38)/cohort	To find a link between complementary feeding and infant gut microbiota composition, diversity and SCFAs.	Early introduction to complementary foods.	Visit at age 3 month: 18/67 Visit at age 12 month: 13/67	T	Total: 11/7	BF ever:14
8a							
8b			Late introduction to complementary foods.	Visit at age 3 month: 47/67 Visit at age 12 month:36/47	T	Total: 33/16	BF ever:39
8c							
9	Edwards et al. (32)/case control	To compare of SCFAs concentration at 2 and 4 weeks of age in babies fed exclusively breast milk or infant formula	Receiving exclusively breastfeeding	Visit at age 2 weeks: 19/47	T	ND	BF:19
9a				Visit at age 4 weeks: 12/47			BF:12
9b			Receiving formula.	Visit at age 2 weeks: 28/47			MF:28
9c				Visit at age 4 weeks: 27/47			MF:27
10	Fleming et al. (53)/RCT	To investigate whether *B. breve BBG-001* affected: intestinal permeability; intestinal microbiota composition and SCFAs synthesis	Receiving placebo.	16/29	ND	ND	ND
11	Heath et al. (33)/case control	To assess whether SCFAs content is linked with sleep in infants.	Receiving standard care.	29/57	T	ND	ND
11a			Feeding themselves from weaning.	29/57			
12	Holscher et al. (54)/RCT	To compare the proportions of fecal bifidobacteria in infants fed formula with or without prebiotics for 6 weeks.	Receiving breastfeeding.	33/105 Visits at age: 0, 3, 6 weeks.	T	ND	BF:33
12a							
12b							
12c			Receiving control formula.	33/105 Visits at age: 0, 3, 6 weeks.			MF:33
12d							
12e							
13	Iszatt et al. (39)/cohort	To investigate whether exposure to toxicants affect SCFAs and gut microbial composition	Norwegian Microbiota Cohort (NoMIC). Not specified.	70/608	PT (*n* = 13) and T (*n* = 57)	13/55	BF:59
14	Kien et al. (40)/case control	To evaluate process of colonic fermentation of carbohydrate to SCFAs	Infants born at 28–32 weeks' gestation and had reached 2–4 weeks' postnatal age receiving formulas: GP (carbohydrate source, 50% lactose-50% glucose polymer) and LAC (containing all the carbohydrate as lactose).	7/15 (LAC)	PT: 28–32	ND	F:7
14a				8/15 (GP)			F:8
15	Kim et al. (55)/RCT	To the potential role for probiotics in the prevention of childhood eczema.	Receiving placebo, no sign of eczema.	7/33	T	0/7	BF:3, MF:3, M:1
16	Knol et al. (56)/RCT	To assess GOS and fructooligosaccharides (FOS) impact on microbiota structure and function.	Receiving breastfeeding.	19/68	T	4/15	BF:11
16a			Receiving standard formula.	19/68		2/16	BF:19
17	Kok et al. (34)/case control	To observe the impact of extensively hydrolyzed (EH) proteins or free amino acids (AA) on intestinal microbiota composition and immune reactivity.	Healthy term infants from birth to 60 days of age	17/75 Visits at age: baseline, 30, 60 day.	T	ND	BF:17
17a							
17b							
18	Kosuwon et al. (57)/RCT	To determine the effect of consuming Young Child Formula (YCF) supplemented with short chain galactooligosaccharides and long chain fructooligosaccharides (scGOS/lcFOS, ratio 9:1) and *B. breve* M-16V on the development of the fecal microbiota in healthy toddlers.	Healthy toddlers	64/129	T	26/38	MF: 64
19	Liu et al. (58)/RCT	To compare the effects of human milk and infant formulas on fecal SCFAs and bacterial composition in human infants.	Receiving breastfeeding.	35/40/120	T	Total: 0/74	BF:35
19a			Receiving standard formula.	37/40/120			MF:37
20	Lund-Blix et al. (41)/Cohort Dahl et al. (27)/Cohort^a^	Investigation of the association between gestational age and gut microbiome.	No antibiotics intake, Norwegian Microbiota Study (NoMIC) cohort	Visit at age 10 day: 74/519	PT (*n* = 15), T (*n* = 59)	Vaginally born only.	BF: 74
20a				Visit at age 4 month: 54/519	PT (*n* = 6), T (*n* = 48)		BF: 54
20b				Visit at age 12 month: 91/519	PT (*n* = 20), T (*n* = 71)		Not BF (formula and solid food): 71
21	Maldonado et al. (59)/RCT	To evaluate the safety of a follow-on formula with *Lactobacillus salivarius* CECT5713 in 6 month old children.	Receiving formula.	40/80 Visits at age: baseline, 3, 6 month.	T	ND	MF:40
21a							
21b							
22	Mentula et al. (60)/RCT	To find differences in the fecal microbial composition and metabolic function and assess the probiotic efficacy in colicky infants.	Healthy term infants at the age 1–6 weeks	9/18	T	3/6	BF:8; MF:1.
23	Midtvedt et al. (49)/Cross-sectional	To observe the development of functionally active intestinal microbiota.	Receiving exclusively breastfeeding up to 6 month of age.	1/17 Visits at age: baseline, 30, 60 day	T	ND	BF:11
23a			Receiving exclusively breastfeeding up to 3 month of age.	8/17 Visits at age: baseline, 30, 60 day			
23b			Receiving exclusively breastfeeding up to 1 month of age.	2/17 Visits at age: baseline, 30, 60 day			
23c			Receiving formula	6/17			MF:6
24	Mohan et al. (61)/RCT	To evaluate the effect of *Bifidobacterium lactis* Bb12 supplementation on body weight, gut fermentation patterns, and immunologic parameters in preterm infants.	Receiving placebo.	32/69 Visits at age: 1, 2, 3 week.	PT:31.27 ± 2.56	29/3	MF:5; HM+HMF: 27.
24a							
24b							
25	Nilsen et al. (42)/cohort	To identify the nutrient utilization and SCFAs production in infants during the first year of life	Not specified.	Visits at age 0: 100/100	T	22/78	BF (3–6 months): 83; BF (6–9 months): 73; BF (9–12 months): 49.
25a				Visits at age 3 month: 100/100			
25b				Visits at age 6 month: 100/100			
25c				Visits at age 12 month:99/100			
26	Nocerino et al. (62)/RCT	To assess the efficacy of *Bifidobacterium animalis subsp. lactis* BB-12^®^ in colicky infants	Exclusively breastfeeding, aged ≤ 7 weeks.	40/80 Visits: enrolment, 28 day of the study.	T	19/21	BF: 40
26a							
27	Nogacka et al. (43)/cohort	To evaluate the impact of intrapartum antimicrobial prophylaxis (IAP) on microbiome	IAP +	18/40 Visits at age: 2, 10, 30, and 90 days.	T	0/40	BF:11; MF:7
27a							
27b			IAP –	22/40 Visits at age: 2, 10, 30, and 90 days.			BF:18; MF:4
27c							
28	Oshiro et al. (63)/RCT	To evaluate the efficacy of Bifidobacterium breve (BBG-01) along with mother's colostrum and breast milk on growth and fecal parameters in preterm infants	Infants born between 24 and 31 weeks of gestation and with body weights <1,500. g	Visit at age 0 week: 15/35	PT:28.2 ± 3.3	15/3	BM: 13; M: 4
28a				Visit at age 1 week: 14/35			
28b				Visit at age 2 weeks: 17/35			
28c				Visit at age 4 weeks: 18/35			
28d				Visit at age 8 weeks: 17/35			
29	Park et al. (44)/cohort	To evaluate the effect of composition and function of gut microbiota at 6 months on atopic dermatitis up to 24 months in early childhood.	Not specified.	84/132	T	25/58	BF:25 MF:10 M:47
30	Pourcyrous et al. (50)/Cross-sectional	Analysis of interactions among GI tract, microbiota, diet and risk of NEC.	Delivery at <32 weeks of gestation. Expreseed maternal breast milk. Preterm formula.	13/32	PT:27.3 ± 2.3	11/2	BM+HMF: 13
30a				19/32	PT:26.6 ± 2.1	10/19	MF:19
31	Quin et al. (35)/case control	Determination if probiotics intake affects infant microbiome, immune markers, SCFA production and health.	Healthy term infants, predominantly breastfed, mother and child without probiotic treatment	52/87	T	15/36	BF: 52
32	Rao et al. (45)/cohort	Comparison of stool microbiota and SCFAs of term infants with congenital gastrointestinal surgical conditions (CGISCs) to healthy control.	Not specified.	Visit at age 1 week: 23/73;	T	12/24	BF: 26
32a				Visit at age 2 week: 17/73.			
33	Roduit et al. (46)/cohort	Analysis of association between fecal SCFA and development of allergic diseases and atopic sensitization later in life.	Children from the Protection against Allergy—Study in Rural Environments (PASTURE).	301	ND	38/263	At 2 months: BF: 184; MF: 45; M: 50.
34	Sierra et al. (64)/RCT	To check the prebiotic effect of GOS on intestinal microbiota to decrease infections and allergy manifestations in healthy infants during the first year of life	Gestational age of 37–42 weeks, birth weight > 2,500 g and were exclusively formula fed for at least 15 days prior to enrolment.	Total: 37/365 Visit at enrolment: 37/365; Visit at age 4 month: 29/365.	T	55/122 (all study group)	MF: 177 (all study group)
34a							
35	Stansbridge et al. (65)/RCT	To assess the efficacy of *Lactobacillus rhamnosus* GG to modify enteric carbohydrate fermentation in preterm infants.	Preterm < 33 gestational weeks	10/20 SCFAs were measured in samples pooled in the following age ranges (days): 1–7; 8–14; 15–21; 22–28; 1–28.	PT: <33	ND	3–10 day of life: BM: 9; MF: 1. Later: all MF.
35a							
35b							
35c							
35d							
36	Ta et al. (47)/cohort	To evaluate the functionality of the gut microbiome and metabolome in eczema.	Not specified.	Visit at age 3 weeks: 13/63;	T	10//20	BF: 2; MF: 16; M: 12.
36a				Visit at age 3 months: 16/63;			
36b				Visit at age 6 months: 27/63;			
36c				Visit at age 12 months: 26/63.			
37	Tauchi et al. (51)/cross-sectional	To elucidate the dynamics and equilibria of the developing microbiota in preterms.	Not specified.	59 Samples were collected at 5 days and approximately 1 month post-delivery.	T (*n* = 42) and PT (*n* = 17) : 34.2 ± 2.7	33/26	Term: BF: 18; M: 22; F: 2 PreTerm: M: 16, BM: 1.
37a							
38	Underwood et al. (66)/RCT	To compare the effect of two prebiotic/probiotic products on weight gain, stool microbiota, and stool SCFA of premature infants.	Birth weight 750–2,000 g, gestational age <35 weeks	29/90	PT:29.3 ± 2.6	23/6	BM: 11; FM: 7; M: 11.
39	Westerbeek et al. (67)/RCT^b^	To determine the effects of enteral supplementation of a prebiotic mixture of neutral and acidic oligosaccharides (scGOS/lcFOS/pAOS) on the fecal microbiota and microenvironment in preterm infants.	Gestational age < 32 weeks and/or birth weight < 1,500 g,	58/113 Samples collected at: <48 h; days: 7, 14, 30.	PT: 31.1 ± 6.4	26/32	BM: 33; FM: 25.
39a							
39b							
39c							
40	Wopereis et al. (69)/RCT	Analysis of the efficacy of amino-acid based formula (AAF) including specific synbiotics on oral and gastrointestinal microbiota of infants with non-IgE mediated cow's milk allergy (CMA).	Receiving formula. Receiving breastfeeding	Visit at week 0: 33/122	T	ND	BF: 51; FM: 36.
40a				Visit at week 8 (controls): 31/122 Visit at week 8 (healthy subjects): 48/122			
40b				Visit at week 12: 26/122			
40c				Visit at week 26: 23/122.			
41	Wopereis et al. (68)/RCT	Th effects of interventions and breast-feeding on fecal microbiota were investigate and link with eczema	Receiving formula. Receiving breastfeeding	57/138 Visits at age: 4, 12, 26 weeks.	ND	11/46	MF:57
41a				30/138 Visits at age: 4, 12 weeks,		5/25	BF:30
41b				51/138 Visits at age: 4, 12 weeks,		10/41	MF (partially hydrolized): 51

*N, number; GOS, galacto-oligosaccharide; ME, median; IQR, interquartile range; IgE, immunoglobulin E; NEC, necrotizing enterocolitis; GI, gastrointestinal; T, term; PT, pre-term; BF, breast fed; BM, Breast Mother Milk; MF, milk formula; SF, soya based formula; HM, Human milk; M, mixed fed; HM + HMF, Human Milk and Human Milk Fortifier.*

a *source of data indicated by Lund-Blix et al. (41)*.

b *source of data (28)*.

### SCFA Concentrations by Time of Analysis (Time After Delivery)

For the meta-analysis, we used data from studies that had only means and standard deviations and where standardization of concentration units was possible. Consequently, pooling was only possible for data obtained up to 9 months of age. The raw data with all abstracted information on concentrations at 3 years of age can be found in the Supplementary Tables 1–4.

**Figure 2:**
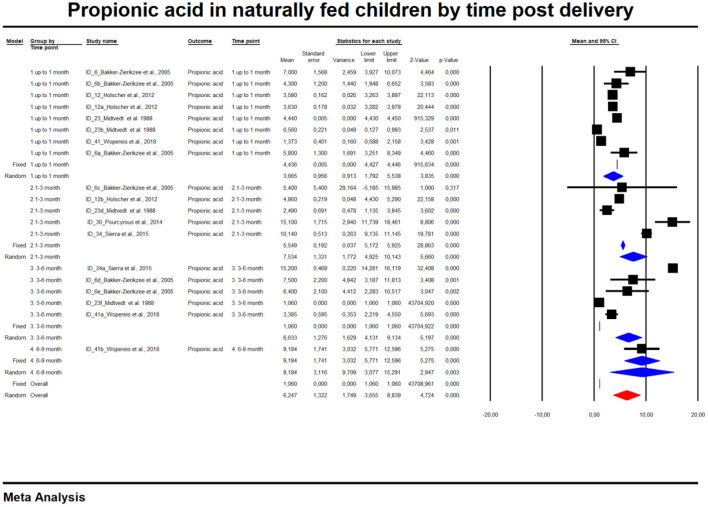
Concentration of propionic acid in naturally fed children by time.

**Figure 3:**
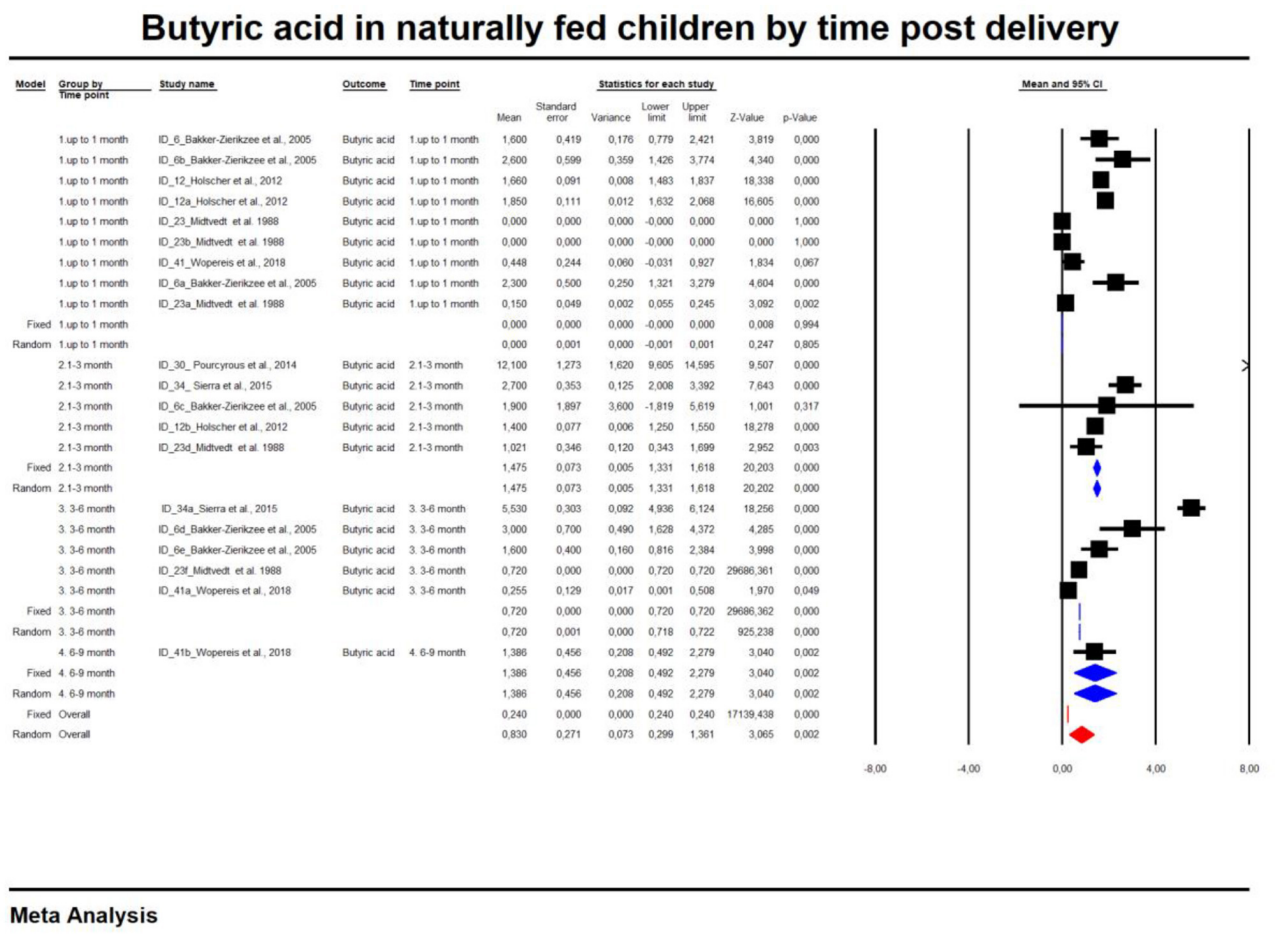
Concentration of butyric acid in naturally fed children by time.

For the sake of clarity, we performed a subgroup analysis by time of evaluation separately for breastfed and formula-fed children. We found that the concentrations of C2, C3, C4 and pooled SCFAs in artificially fed children did not differ by time of evaluation (*p* > 0.05). In contrast, the concentrations of propionate and butyrate in breastfed infants differ significantly with respect to the time period after birth (Figures 2, 3). Table 2 also details the concentrations (mean, standard deviation and ranges) of C2, C3, and C4 at different life stages in relation to feeding method.

**Table 2:** The mean concentrations of SCFAs (μmol/g) by nutrition method and time range after birth.

	**Time point**	**Number** **of studies**	**Effect** **size (mean)**	**SE**	**Variance**	**Lower** **limit**	**Upper** **limit**	**Test Z** **(z value)**	**Test Z (*p*-value)**	**Q value**	**df (Q)**	***p*-value**	**heterogenity** **(I2 from fixed** **effect** **analysis)**
	**Acetic acid**												
**Formula-fed**	Up to 1 month	7	50.45	9.12	83.23	32.56	68.33	5.53	0.00	1,791.94	6.00	0.00	99.67
	1–3 months	5	54.37	10.86	117.91	33.09	75.65	5.01	0.00	382.12	4.00	0.00	98.95
	3–6 months	2	62.91	17.29	299.11	29.01	96.81	3.64	0.00	3.60	1.00	0.06	72.21
	Total between									0.41	2.00	0.81	
	Overall	14	53.59	6.48	41.95	40.89	66.28	8.27	0.00	2,449.03	13.00	0.00	99.47
	**Propionic acid**												
	Up to 1 month	7	12.23	2.33	5.41	7.67	16.79	5.26	0.00	1,639.5478	6.00	0.00	99.63
	1–3 months	5	13.97	2.78	7.73	8.52	19.42	5.03	0.00	31.6183312	4.00	0.00	87.35
	3–6 months	2	15.83	4.52	20.39	6.98	24.68	3.50	0.00	4.70742213	1.00	0.03	78.76
	Total between									0.58	2.00	0.75	
	Overall	14	13.34	1.66	2.75	10.08	16.59	8.04	0.00	2,049.4582	13.00	0.00	99.37
	**Butyric acid**												
	Up to 1 month	6	3.30	1.02	1.03	1.30	5.29	3.24	0.00	662.404243	5.00	0.00	99.25
	1–3 months	5	5.16	1.16	1.34	2.90	7.43	4.47	0.00	10.1095259	4.00	0.04	60.43
	3–6 months	2	5.48	1.87	3.50	1.81	9.14	2.93	0.00	2.85E-02	1.00	0.87	0.00
	Total between									1.93	2.00	0.38	
	Overall	13	4.40	0.89	0.79	2.66	6.14	4.96	0.00	934.66	12.00	0.00	98.72
	**All SCFAs (not specified)**												
	Up to 1 month	5	55.92	4.84	23.41	46.44	65.40	11.56	0.00	42.54	4.00	0.00	90.60
	1–3 months	4	62.10	5.92	35.10	50.49	73.71	10.48	0.00	11.52	3.00	0.01	73.96
	3–6 months	1	68.60	16.47	271.26	36.32	100.88	4.17	0.00	0.00	0.00	1.00	0.00
	total between									1.02	2.00	0.60	
	Overall	10	58.91	3.68	13.56	51.69	66.13	16.00	0.00	55.97	9.00	0.00	83.92
	**Acetic acid**												
	Up to 1 month	8	50.04	9.13	83.40	32.14	67.94	5.48	0.00	4,345.05	7.00	0.00	99.84
**Breast fed**	1–3 months	5	52.26	11.62	135.01	29.49	75.03	4.50	0.00	2,740.10	4.00	0.00	99.85
	3–6 months	5	67.16	11.53	132.85	44.57	89.75	5.83	0.00	1,968.54	4.00	0.00	99.80
	6–9 months	1	59.50	25.99	675.52	8.56	110.44	2.29	0.02	0.00	0.00	1.00	0.00
	Total between									1.48	3.00	0.69	
	Overall	19	55.88	6.56	43.00	43.03	68.73	8.52	0.00	14,431.66	18.00	0.00	99.88
	**Propionic acid**												
	Up to 1 month	8	3.66	0.96	0.91	1.79	5.54	3.83	0.00	420.24	7.00	0.00	98.33
	1–3 months	5	7.53	1.33	1.77	4.93	10.14	5.66	0.00	140.69	4.00	0.00	97.16
	3–6 months	5	6.63	1.28	1.63	4.13	9.13	5.20	0.00	939.09	4.00	0.00	99.57
	6–9 months	1	9.18	3.12	9.71	3.07	15.29	2.95	0.00	0.00	0.00	1.00	0.00
	Total between									8.19	3.00	0.04	
	Overall	19	6.25	1.32	1.75	3.65	8.84	4.72	0.00				100.00
	**Butyric acid**												
	Up to 1 month	9	0.01	0.01	0.00	0.00	0.02	2.42	0.02	679.59	8.00	0.00	98.82
	1–3 months	5	1.48	0.07	0.01	1.33	1.62	20.12	0.00	84.44	4.00	0.00	95.26
	3–6 months	5	0.72	0.01	0.00	0.71	0.74	93.00	0.00	280.73	4.00	0.00	98.58
	6–9 months	1	1.39	0.46	0.21	0.50	2.28	3.04	0.00	0.00	0.00	1.00	0.00
	Total between									5,869.80	3.00	0.00	
	Overall	20	0.84	0.27	0.07	0.30	1.37	3.08	0.00	5,876,411.12	19.00	0.00	100.00
	**All SCFAs (not specified)**												
	Up to 1 month	5	45.28	6.86	47.02	31.84	58.72	6.60	0.00	63.13	4.00	0.00	93.66
	1–3 months	3	71.30	9.22	85.06	53.22	89.38	7.73	0.00	113.98	2.00	0.00	98.25
	3–6 months	2	59.83	11.33	128.32	37.63	82.03	5.28	0.00	0.02	1.00	0.89	0.00
	Total between									5.30	2.00	0.07	
	Overall	10	57.98	9.23	85.23	39.89	76.08	6.28	0.00	220.12	9.00	0.00	95.91

**Figure 4:**
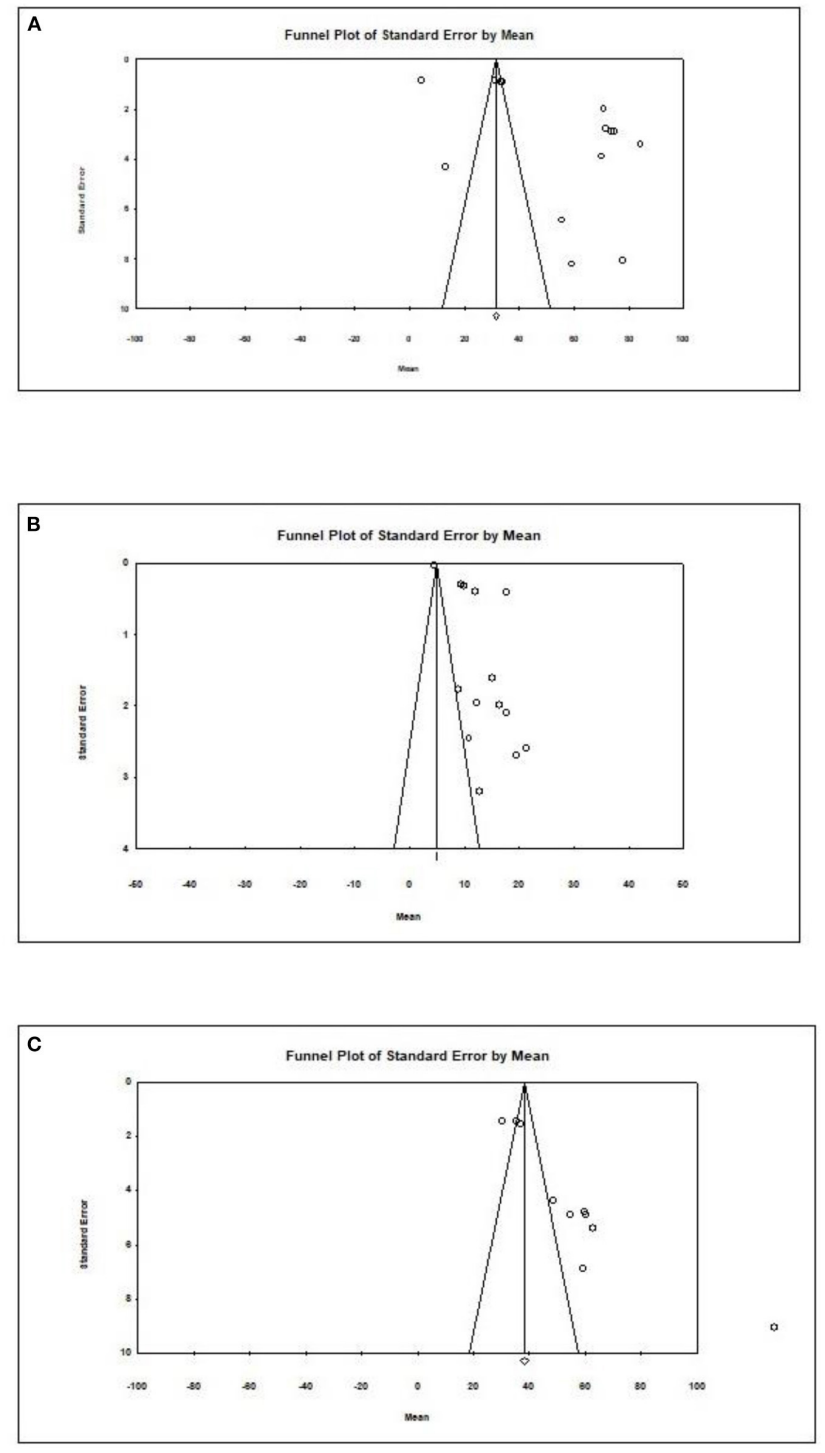
Publication bias for different outcomes in present metaanalysis. **(A)** Acetic acid/Formula. **(B)** Propionic acid/Formula. **(C)** All SCFAs/Breast.

Overall, an Egger test does not indicate publication bias in all but three cases (Figure 4) (Egger test: *p* > 0.05). However, the Duval and Tweedie method adjusted values in only one case; 1 study to the left of the mean was truncated for propionic acid in formula-fed infants (point estimate of random model: 4.8622; 95% CI: 4.7881–4.9364, Q value = 2,049.520).

In the final part of our study, we analyzed data on SCFAs other than those previously described. The results are presented in Table 3.

**Table 3:** The concentrations of other SCFAs (μmol/g) in healthy children by time.

**References**	**Study ID**	**Time range**	**Time when evaluated**	**Time unit when evaluate day (day/week/** **month)**	**Other SCFA name**	**Concentration (Mean)**	**Concentration (SD)**	**Concentration unit**
Bakker-Zierikzee et al. (31)	6	**UP TO 1 MONTH**	5	Day	iC4-5 SCFA. sum of isobutyrate. isovalerate and valerate;	2	2.37	μmol/g
	6a		10	Day		2.6	2.3	
	6b		4	Week		2.1	2.11	
	6f		5	Day		1.1	1.39	
	6g		10	Day		3.2	12.39	
	6h		4	Week		5.5	9	
Edwards et al. (32)	9		2	Week	Isovaleric acid	*M 2.1	*R 0–153.0	μmol/g
	9a		2	week		*M 6.1	*R 0–13.8	
	9b		4	Week		*M 2.1	*R 0–3.5	
	9c		4	Week		*M 6.3	*R 0–17.7	
Fleming et al. (53)	10		21	Day	Lactic acid	*M 4.6	IQR 3.65–5.12	mmol/g
Holscher et al. (54)	12		0	Week	Valeric acid	2.06	1.74	μmol/g
	12a		3	Week		1.41	1.37	
	12c		0	Week		2.38	1.91	
	12d		3	Week		1.46	1.13	
Iszatt et al. (39)	13		1	Month	Isovaleric acid	0.28	0.41	μmol/g
Kien et al. (40)	14 (I)		2–4	Week	Isobutyric acid	0.01	0.016	kcal/day
	14 (II)		2–4	Week	Isovaleric acid	0.03	0.017	
	14 (III)		2–5	Week	Valeric acid	0	0.007	
Nilsen et al. (42)	25		0	Month	All other (isobutyrate. isovalerate. valerate)	9.8	nd	%
Lund-Blix et al. (41)	20 (I)		10	Days	Isobutyric acid	PT: *M 0.0 T:*M 0.0	PT: *R 0.0–0.07 T:*R 0.0–0.14	μmol/g
	20 (II)		10	Days	Isovaleric acid	PT: *M 0.1 T:*M 0.04	PT: *R 0.0–0.26 T:*R 0.0–0.12	
	20 (III)		10	Days	Valeric acid	PT: *M 0.0 T:*M 0.0	PT: *R 0.0–0.08 T:*R 0.0–0.04	
	20 (IV)		10	Days	Isocaproic acid	PT: *M 0.0 T:*M 0.0	PT: *R 0.0–0.0 T:*R 0.0–0.0	
	20 (V)		10	days	Caproic acid	PT: *M 0.0 T:*M 0.0	PT: *R 0.0–0.0 T:*R 0.0–0.1	
Wopereis et al. (68)	41 (I)		4	Week	Valeric acid	0.0086	2.79397E-10	μmol/g
	41 (II)		4	Week	Isobutyric acid	0.0086	2.79397E-10	
	41 (III)		4	Week	Isovaleric acid	0.0086	2.79397E-10	
Brink et al. (36)	4	**1–3 MONTHS**	3	Month	Valeric acid	10.8 (*M. positive mode)	2.08 (IQR)	log transformed peak areas
	4d		3	Month		13.2(*M. positive mode)	4.18 (IQR)	
	4h		3	Month		(*M. positive mode)	13.1 (IQR)	
Bakker-Zierikzee et al. (31)	6c		8	Week	iC4-5 SCFA. sum of isobutyrate. isovalerate and valerate;	1.6	7.5	μmol/g
	6i		8	Week		2.9	2.62	
Differding et al. (38)	8		3	Month	Other SCFA NAme (*Low abundance SCFAs including isobutyric acid. valeric acid. isovaleric acid. heptanoic acid. and hexanoic acid have been grouped into the “other” category for visual clarity)	4.4	nd	%
	8b		3	Month		3.9	nd	
Holscher et al. (54)	12b		6	Week	Valeric acid	0.97	0.87	μmol/g
	12e		6	Week		1.23	0.98	
Nilsen et al. (42)	23a		3	Month	All other (isobutyrate. isovalerate. valerate)	5.6	nd	%
Pourcyrous et al. (50)	30, 30a		first 80 days of life	Day	Isobutyric acid	N: 20.82 A:10.6	N:25.2 A:23.5	μmol/g
Brink et al. (36)	4a	**3–6 MONTHS**	6	Month	Valeric acid	10.5 (*M. positive mode)	2.14 (IQR)	log transformed peak areas
	4e		6	Month		15.3 (*M. positive mode)	2.21 (IQR)	
	4i		6	Month		13.2 (*M. positive mode)	2.4 (IQR)	
Bakker-Zierikzee et al. (31)	6d		12	Week	iC4-5 SCFA. sum of isobutyrate. isovalerate and valerate;	3.5	3.9	μmol/g
	6j		12	Week		3.2	1.87	
	6e		16	Week		2.2	2.06	
	6k		16	Week		4.9	2.77	
Nilsen et al. (42)	23b		6	Month	All other (isobutyrate. isovalerate. valerate)	1.7	nd	%
Lund-Blix et al. (41)	20a (I)		4	Month	Isobutyric acid	PT: *M 1.6 T:*M 0.7	PT: *R 1.2–3.0 T:*R 0.3–1.4	μmol/g
	20a (II)		4	Month	Isovaleric acid	PT: *M 1.7 T:*M 0.7	PT: *R 1.5–3.5 T:*R 0.09–1.9	
	20a (III)		4	Month	Valeric acid	PT: *M 0.08 T:*M 0.05	PT: *R 0.0–0.5 T:*R 0.0–0.11	
	20a (IV)		4	Month	Isocaproic acid	PT: *M 0.06 T:*M 0.0	PT: *R 0.0–0.4 T:*R 0.0–0.0	
	20a (V)		4	Month	Caproic acid	PT: *M 0.0 T:*M 0.0	PT: *R 0.0–0.0 T:*R 0.0–0.06	
Wopereis et al. (68)	41a (I)		12	Week	Valeric acid	0.0086	2.6885E-10	μmol/g
	41a (II)		12	Week	Isobutyric acid	0.061600422	0.265002108	
	41a (III)		12	Week	Isovaleric acid	0.21198658	0.487114382	
Brink et al. (36)	4b		9	Month	Valeric acid	13.3 (*M. positive mode)	2.51 (IQR)	log transformed peak areas
	4f		9	Month		13.1 (*M. positive mode)	1.81 (IQR)	
	4j		9	Month		14.6 (*M. positive mode)	3.25 (IQR)	
Wopereis et al. (68)	41b (I)	**6–9 MONTHS**	26	Week	Valeric acid	0.054087559	0.24914562	μmol/g
	41b (II)		26	Week	Isobutyric acid	0.348646926	0.753113276	
	41b (III)		26	Week	Isovaleric acid	0.525732305	0.885977824	
Brink et al. (36)	4c		12	Month	Valeric acid	13.9 (*M. positive mode)	2.54 (IQR)	log transformed peak areas
	4g		12	Month		16.4 (*M. positive mode)	3.65(IQR)	
	4k		12	Month		16 (*M. positive mode)	3.3 (IQR)	
	4l		12	Month		14.9 (*M. positive mode)	2.64 (IQR)	
Differding et al. (38)	8a	**9–12 MONTHS**	12	Month	Other SCFA NAme (*Low abundance SCFAs including isobutyric acid. valeric acid. isovaleric acid. heptanoic acid. and hexanoic acid have been grouped into the “other” category for visual clarity)	3.4	nd	%
	8c		12	Month		4.9	nd	
Nilsen et al. (42)	25c		12	Month	All other (isobutyrate. isovalerate. valerate)	2.5	nd	%
Díaz et al. (37)	7	**>12 MONTHS**	12–24	Month	Branched chain fatty acids (BCFAs). isobutyric and isovaleric acids	*MA: 2.59	IQR: 1.94–3.37	μmol/g
Kosuwon et al. (57)	18 (I)		18 (I)	18 (I)	Valeric acid	*M 2.18	*R 0.08–3.12	μmol/g
	18 (II)		18 (II)	18 (II)	Isobutyric acid	*M 2.46	*R 1.75–3.55	
	18 (III)				Isovaleric acid	*M 3.26	*R 2.17–5.28	
Lund–Blix et al. (41)	20b (I)		12	Month	Isobutyric acid	PT: *M 1.8 T:*M 1.55	PT: *R 0.9–2.3 T:*R 0.82–2.42	μmol/g
	20b (II)		12	Month	Isovaleric acid	PT: *M 2.3 T:*M 1.96	PT: *R 0.6–3.2 T:*R 0.92–2.99	
	20b (III)		12	Month	Valeric acid	PT: *M 0.18 T:*M 0.25	PT: *R 0.03–0.27 T:*R 0.07–0.82	
	20b (IV)		12	Month	Isocaproic acid	PT: *M 0.0 T:*M 0.0	PT: *R 0.0–0.0 T:*R 0.0–0.0	
	20b (V)		12	Month	Caproic acid	PT: *M 0.0 T:*M 0.0	PT: *R 0.0–0.0 T:*R 0.0–0.0	

PT, pre-term, T-term; ^*^ R, range; ^*^IQR, interquartile range; ^*^M, median.

### SCFA Concentrations by Feeding Time

**Figure 5:**
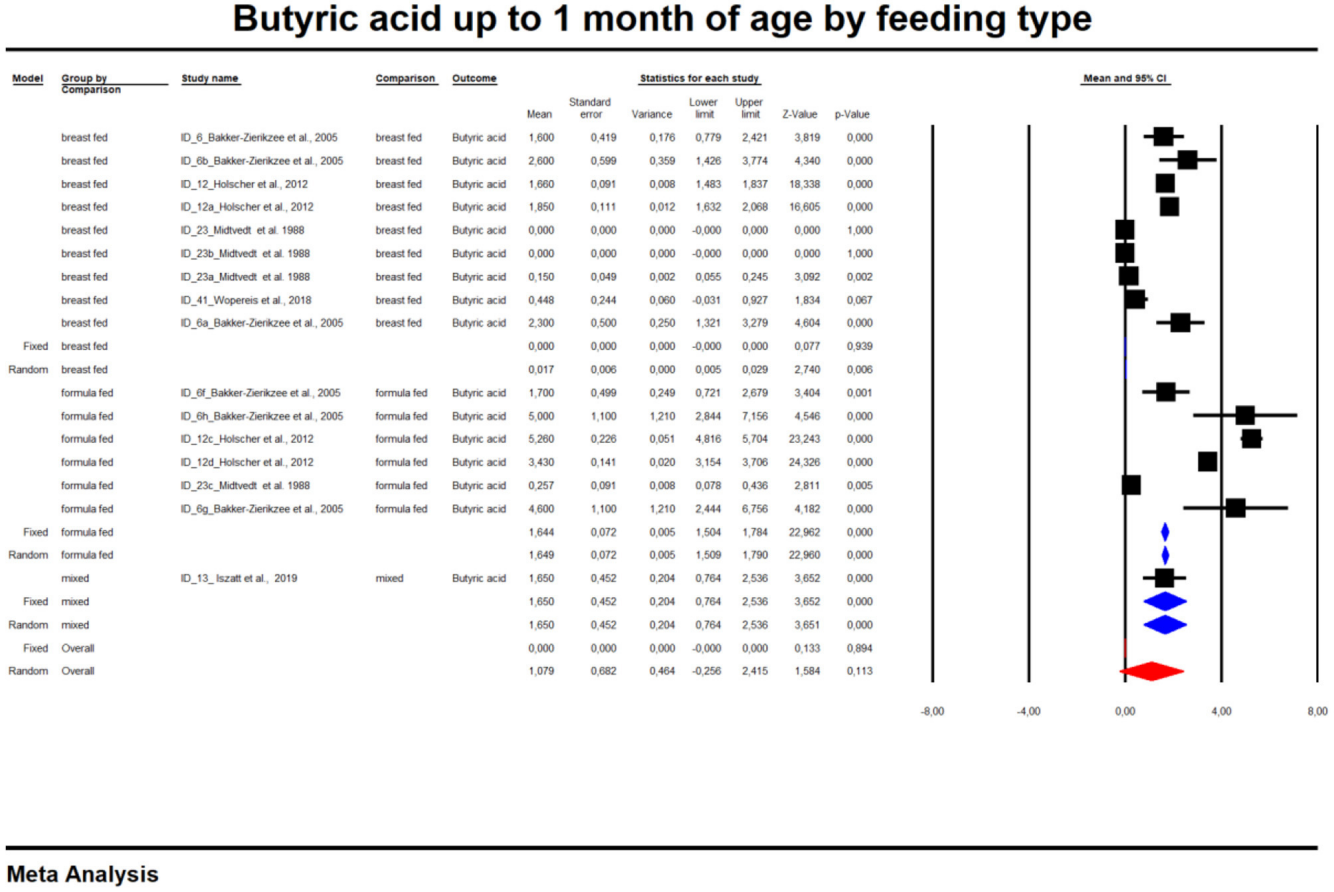
Concentration of butyric up to 1 month of age by feeding type.

**Figure 6:**
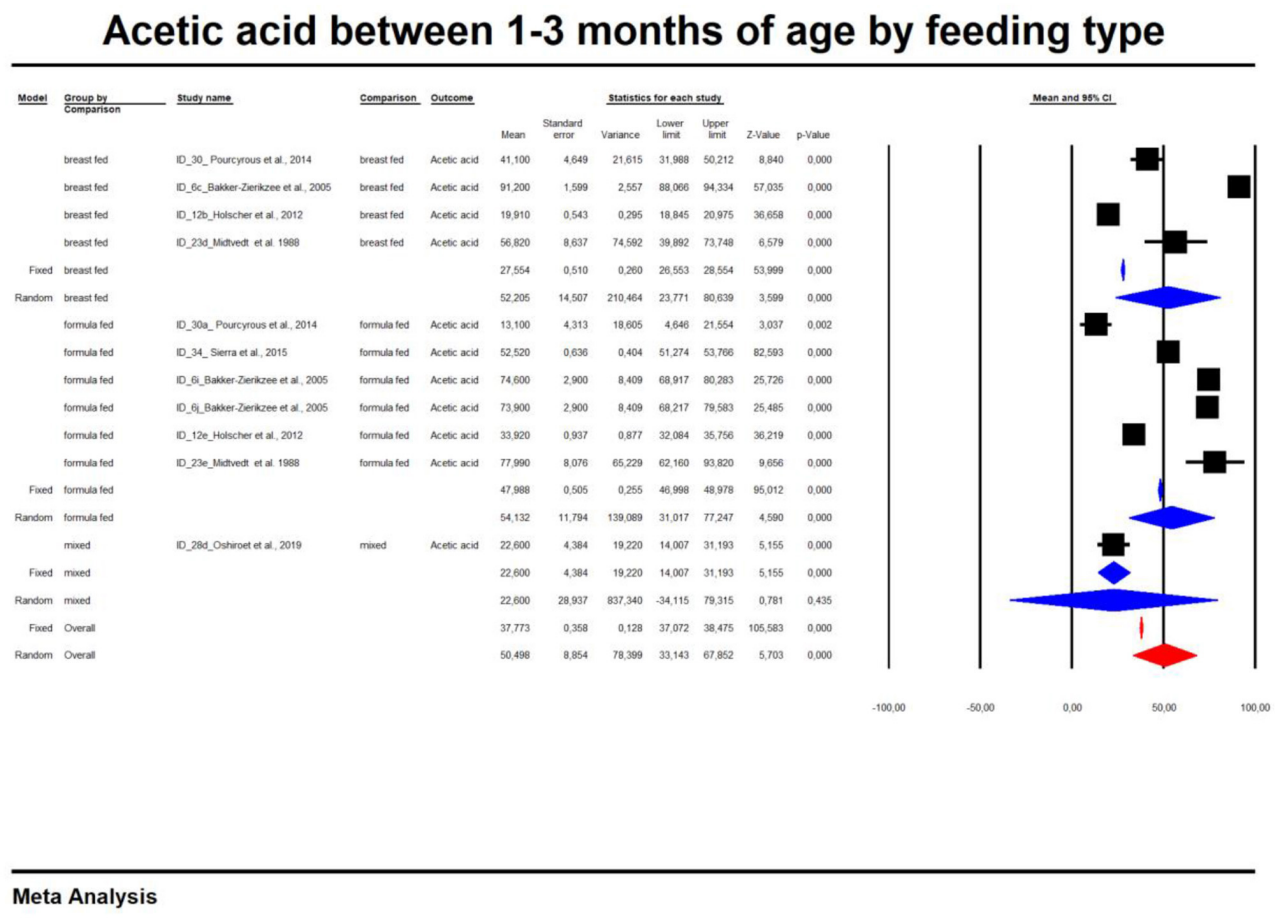
Concentration of acetic between 1 and 3 month of age by feeding type.

In the last part of our analyses, we decided to analyse how diet was related to the levels of SCFAs in specific age groups. In this case, pooling synthesis was possible due to data availability as well as the units to be standardized for children up to 6 months of age. We found that in the vast majority of infants, formula feeding significantly increased the level of SCFAs. In infants up to 1 month of age who are artificially fed, the concentration of propionic acid, butyric acid and all SCFAs is higher and that of acetic acid is lower. At 1–3 months of age, a higher concentration of only propionic acid was observed. At 3–6 months of age, artificial feeding leads to a higher concentration of butyric acid and the sum of SCFAs. The results are shown in Table 4 and Figures 5, 6. In one case—up to 1 month, butyric acid—we found that Egger's test indicated publication bias. Using the method of Duval and Tweedie, 8 studies were trimmed to the left of the mean, with a point estimate of the random-effects model: 0.028; 95% CI: 0.009–0.046, Q value = 3,373.605.

**Table 4:** Effect sizes (Means) for SCFAs (μmol/g) in particular age ranges by feeding type.

	**Time point**	**Number** **of studies**	**Effect** **size (mean)**	**SE**	**Variance**	**Lower** **limit**	**Upper** **limit**	**Test Z** **(z value)**	**Test Z (*p*-value)**	**Q value**	**df (Q)**	***p*-value**	**heterogenity** (I2*from fixed effect **analysis)**
	**Acetic acid**												
	Breast fed	8	50.05	5.84	34.12	38.60	61.50	8.57	0.00	4,345.04	7.00	0.00	99.84
**Up to 1 month**	Formula fed	6	46.57	6.78	45.92	33.29	59.85	6.87	0.00	1,305.58	5.00	0.00	99.62
	Mixed	8	22.39	5.86	34.33	10.91	33.87	3.82	0.00	217.01	7.00	0.00	96.77
	Total between									12.86	2.00	0.00	
	Overall	22	39.57	9.34	87.26	21.26	57.88	4.24	0.00	5,896.88	21.00	0.00	99.64
	**Propionic acid**												
	Breast fed	8	3.69	1.06	1.13	1.61	5.77	3.48	0.00	419.90	7.00	0.00	98.33
	Formula fed	7	11.83	1.21	1.47	9.45	14.21	9.75	0.00	1,639.55	6.00	0.00	99.63
	Mixed	2	3.39	2.08	4.34	−0.69	7.48	1.63	0.10	3.18	1.00	0.07	68.59
	Total between									28.41	2.00	0.00	
	Overall	17	6.39	3.04	9.27	0.42	12.36	2.10	0.04	2,146.24	16.00	0.00	99.25
	**Butyric acid**												
	Breast fed	9	0.02	0.01	0.00	0.00	0.03	2.74	0.01	679.57	8.00	0.00	98.82
	Formula fed	6	1.65	0.07	0.01	1.51	1.79	22.96	0.00	662.40	5.00	0.00	99.25
	Mixed	1	1.65	0.45	0.20	0.76	2.54	3.65	0.00	0.00	0.00	1.00	0.00
	Total between									525.49	2.00	0.00	
	Overall	16	1.08	0.68	0.46	−0.26	2.42	1.58	0.11	1,882.53	15.00	0.00	99.20
	**All SCFAs (not specified)**												
	Breast fed	5	44.55	4.30	18.51	36.11	52.98	10.35	0.00	63.13	4.00	0.00	93.66
	Formula fed	5	55.99	4.94	24.42	46.31	65.68	11.33	0.00	42.54	4.00	0.00	90.60
	Total between									3.05	1.00	0.08	
	Overall	10	50.01	5.72	32.68	38.81	61.22	8.75	0.00	232.55	9.00	0.00	96.13
	**Acetic acid**												
	Breast fed	5	52.27	12.59	158.43	27.60	76.94	4.15	0.00	2,740.10	4.00	0.00	99.85
**1-3 months**	Formula fed	5	54.45	12.59	158.58	29.77	79.13	4.32	0.00	382.12	4.00	0.00	98.95
	Mixed	1	22.60	28.15	792.46	−32.57	77.77	0.80	0.42	0.00	0.00	1.00	0.00
	Total between									1.10	2.00	0.58	
	Overall	11	50.40	8.79	77.29	33.17	67.64	5.73	0.00	3,144.24	10.00	0.00	99.68
	**Propionic acid**												
	Breast fed	5	7.67	1.81	3.28	4.12	11.22	4.24	0.00	140.69	4.00	0.00	97.16
	Formula fed	5	13.87	1.82	3.31	10.30	17.43	7.63	0.00	31.62	4.00	0.00	87.35
	Mixed	1	3.20	3.69	13.59	−4.03	10.43	0.87	0.39	0.00	0.00	1.00	0.00
	Total between									9.58	2.00	0.01	
	Overall	11	8.79	3.02	9.10	2.88	14.71	2.92	0.00	406.16	10.00	0.00	97.54
	**Butyric acid**												
	Breast fed	5	3.28	0.76	0.57	1.79	4.76	4.33	0.00	84.44	4.00	0.00	95.26
	Formula fed	5	5.08	0.78	0.61	3.55	6.60	6.51	0.00	10.11	4.00	0.04	60.43
	Total between									2.73	1.00	0.10	
	Overall	10	4.17	0.90	0.81	2.41	5.93	4.64	0.00	362.79	9.00	0.00	97.52
	**All SCFAs (not specified)**												
	Breast fed	3	74.58	17.44	304.05	40.40	108.75	4.28	0.00	113.98	2.00	0.00	98.25
	Formula fed	4	66.48	15.56	241.97	35.99	96.97	4.27	0.00	11.52	3.00	0.01	73.96
	Total between									0.12	1.00	0.73	
	Overall	7	70.07	11.61	134.74	47.32	92.82	6.04	0.00	151.19	6.00	0.00	96.03
	**Acetic acid**												
	Breast fed	5	67.19	9.56	91.42	48.45	85.93	7.03	0.00	1,968.54	4.00	0.00	99.80
	Formula fed	2	62.95	15.45	238.61	32.67	93.22	4.08	0.00	3.60	1.00	0.06	72.21
	Total between									0.05	1.00	0.82	
	Overall	7	66.01	8.13	66.09	50.08	81.95	8.12	0.00	2,007.52	6.00	0.00	99.70
	**Propionic acid**												
	Breast fed	5	6.70	3.50	12.27	−0.17	13.56	1.91	0.06	939.19	4.00	0.00	99.57
	Formula fed	2	15.88	5.70	32.48	4.71	27.05	2.79	0.01	4.71	1.00	0.03	78.76
**3-6 months**	Total between									1.89	1.00	0.17	
	Overall	7	10.19	4.46	19.88	1.45	18.93	2.29	0.02	1,019.54	6.00	0.00	99.41
	**Butyric acid**												
	Breast fed	5	2.16	0.61	0.37	0.96	3.36	3.54	0.00	280.55	4.00	0.00	98.57
	Formula fed	2	5.49	1.21	1.46	3.12	7.85	4.55	0.00	0.03	1.00	0.87	0.00
	Total between									6.03	1.00	0.01	
	Overall	7	3.66	1.65	2.73	0.42	6.90	2.21	0.03	322.66	6.00	0.00	98.14
	**All SCFAs (not specified)**												
	Breast fed	5	2.16	0.61	0.37	0.96	3.36	3.54	0.00	280.55	4.00	0.00	98.57
	Formula fed	2	5.49	1.21	1.46	3.12	7.85	4.55	0.00	0.03	1.00	0.87	0.00
	Total between									6.03	1.00	0.01	
	Overall	7	3.66	1.65	2.73	0.42	6.90	2.21	0.03	322.66	6.00	0.00	98.14

## Discussion

This paper is the systematic review aimed at analyzing SCFAs over 2 years of life and the influence of feeding method on the content of these compounds in feces. However, based on the available data, only the concentration of fecal SCFAs in healthy children in the first year of life was meta-analyzed. We also presented detailed data on the concentration of SCFAs in feces by life span of the newborn (up to 1 month, 3–6 months and 6–9 months old), which can serve as a reference for other researchers. We have shown that breastfeeding is associated with changes in propionate and butyrate concentrations in the first 9 months of life and that this effect is not observed in children fed with artificial infant formula. On the other hand, a higher concentration of SCFAs (propionic acid, butyric acid, all SCFAs) in the stool is observed in artificially fed children compared to breastfed children at different stages of life. Only in the first month of life is the concentration of acetic acid lower in artificially fed children.

The content of SCFAs in the stool is the result of a trade-off between the production of these compounds by the intestinal bacteria, their uptake and their consumption *in situ*, in the gastrointestinal tract. Due to the numerous important metabolic functions performed by SCFAs during development and the impossibility (for ethical reasons) of determining their concentration in the blood of healthy newborns, the concentration of SCFAs in stool is an indirect marker of their content in the body. This meta-analysis includes a comparative evaluation of the effects of breastfeeding on the concentration of SCFAs in the stool of children up to 9 months of age, i.e., during a period when solid food is either not introduced into the diet or only introduced to a small extent.

The meta-analysis compared the effect of diet on the stool content of the three most important SCFAs, namely acetic acid, propionic acid and butyric acid, with the sum of SCFAs. The content of other acids could not be included in the meta-analysis because there was insufficient data for such a synthesis. The observed changes in the concentration of SCFAs in feces could be related to changes in the composition and function of the microbiota during the first phase of life. Acetic acid is the most abundant SCFA in the gut, its content being twice that of propionic and butyric acids (70, 71). It is produced by most of the anaerobic bacteria inhabiting the gut; for example, *Bacteroides* spp. and *Akkermansia muciniphila* produce acetic and propionic acids (71, 72). Bacteria of the genus *Clostridioides* produce three important SCFAs (73, 74) of which butyric acid is most important for maintaining a healthy gut. Organisms such as *Anaerostipes, Clostridioides, Coprococcus, Dorea, Eubacterium, Faecalibacterium, Roseburia* and *Ruminococcus* produce butyric acid, the latter three being the most abundant in the gut (75, 76). *Eubacterium and Anaerostipes* interact with *Bifidobacterium* to increase their ability to produce butyric acid. *Bifidobacterium* is involved in the production of butyric acid by producing acetic acid through the fermentation of carbohydrates. Subsequently, the acetic acid is converted to butyric acid by *Eubacterium* and *Anaerostipe*s through a process known as cross-feeding (77). These properties of infant bacteria, *B. bifidum* and *B. breve*, are taken over by adult *Bifidobacterium* strains and by the end of the second year of life the pattern of adult SCFA production is achieved (16). This confirms the importance of this early period in shaping the metabolism of these compounds. Furthermore, *B. bifidum* is able to produce acetic acid by fermenting mucins (78). Only *A. muciniphila* and *Verrucomicrobium* have similar properties (79).

The data we have presented confirm that changes in the composition of the microbiota in the first phase of life can influence the later production of SCFAs. After birth, the child's microbiota matures and resembles the adult type by 2–3 years of age (80). Characteristics at each stage of development are consistent with the functional maturation of the microbiome. The type of diet has a major influence on the composition and functioning of the microbiota, but it is the cessation of breastfeeding rather than the introduction of solid foods that determines its maturation (1). Our meta-analysis provides evidence that propionic and butyric acid levels change over time only in breastfed infants. The source of gut microbes in infants is the mother's skin, vagina, stool and breastfeeding by the mother (8, 81–83).

The gut microbiota of infants has been shown to differ between breastfed and formula-fed infants (84–89) and changes rapidly during the transition from breastfeeding to formula-fed feeding (90). Microbiota changes in the first 6 months of life were observed by Ho et al. who conducted a meta-analysis of seven studies examining microbiota changes (1,825 stool samples, 684 infants) under the influence of feeding (7). In the first 6 months of life, the diversity of intestinal bacteria, the age of the microbiota indicating its maturity, the relative abundance of Bacteroidetes and Firmicutes, and the predicted pathways of carbohydrate metabolism are greater in artificially fed infants. An increase in the relative abundance of Bacteroidetes and Firmicutes and of bacteria at lower taxonomic levels was observed in artificially fed infants (order: *Bacteriodales, Clostridiale*, families: *Bacteroidaceae, Veillonellaceae*; genera: *Bacteroides, Eubacterium, Veillonella* and *Megasphaera*) compared to breastfed infants (91) and/or a tendency toward increased bacterial diversity in infants, regardless of the type of diet (92–94). These observations could explain why infants fed with artificial infant formula have higher levels of SCFAs in their stools. However, it should be emphasized that the studies we included in our work were characterized by considerable heterogeneity, which also explains the high heterogeneity of the results of our meta-analysis. It is reasonable to assume that a more stable, less differentiated gut microbiota is present in the context of breastfeeding during the first months of development (7) and that changes in the microbiota caused by artificial feeding may affect children's health later in life. While *Bifidobacterium* is the most common bacterial species in the digestive tract of infants, *Bacteroides* and *Eubacterium* are the most common bacterial species in the intestines of adults (95, 96). While these genera may be part of the normal environment of gut bacteria, an increase in *Bacteroides* in the gut has been shown to be associated with higher body mass index (BMI) in young children (97), while *Veillonella* may be associated, at least to some extent, with various types of infections (98). The increase in the relative abundance of major metabolic pathways related to carbohydrate metabolism in infants fed artificial infant formula and the decrease in the relative abundance of major metabolic pathways related to lipid metabolism/homeostasis, free radical detoxification, and cofactor and vitamin metabolism, may also be associated with a higher risk of obesity, diabetes and other adverse health outcomes in children who were not breastfed early (99–101). From our perspective, the results of the Ho et al. meta-analysis are particularly important because it included children from different geographic regions, which may have had an impact on the microbiota (7).

Observations on the relationship between microbiota composition and stool SCFAs concentration are scarce. Tsukuda et al. (102) observed in 12 children during the first 2 years of life three distinct phases of progression of SCFA profiles: the early phase with a low acetate content and high succinate content, the intermediate phase with a high lactate and formate content, and the late phase with a high propionate and butyrate content. The assessment of the relationship between SCFA and the microbiota showed that the presence of butyrate in the feces is associated with an increased number of *Clostridiales* and the cessation of breastfeeding and that a diverse and individualized set of *Clostridiale*s species harboring acetyl-CoA pathway plays a significant role in the production of butyrates in the intestine. The early microbiome showed significant variation in composition and progression and varied over time. *Enterobacterales* typically dominated the newborn microbiota, but their numbers declined with age. *Bifidobacteriales* constituted the main bacterial order in early-life microbiota, showing an increasing abundance up to 6 months of age and a decrease after 8 months of age. *Clostridiales* were less numerous until 8 months of age and then increased. It was also confirmed that α-diversity increased with age. At the end of the study period, such elevation rates slowed down, although, in 2-year-old infants, this index was still significantly lower than for their parents. The day of transition showed significant inter-individual variability; the transition from *Enterobacterales* to *Bifidobacteriales* lasted from 3 days to 6 months (median, 0.6 months), while the transition time from *Bifidobacteriales* to *Clostridiales* ranged between 8 and 24 months (median, 13 months). Consistent with previous studies (2, 103), the transition from a *Bifidobacteriales*-dominated microbiota to *Clostridiales*-dominated one has been associated with cessation of breastfeeding. These observations explain the previously mentioned heterogeneity of the observed results. As with the gut microbiota development, intestinal SCFAs synthesis profiles during early life were dynamic and individualized, with patterns showing temporal trajectories.

The major SCFA throughout the study period was acetate; its concentration increased to 6 months and then remained at a constant level. The concentration of propionate and butyrate increased after 8 and 10 months of age, respectively. Branched-chain fatty acids (i.e., isobutyrate and isovalerate) were rarely detected throughout the study (especially up to 9 months of age). The authors divided the SCFA pattern into three types, characterized by either low acetates and high succinate (SCFA type 1), high lactate and high formate (SCFA type 2), or high propionate and high butyrate (SCFA type 3). They then summarized the progression of the SCFA type production and observed sequential shifts from SCFA types 1 to 2 and then to type 3, with individual variability on the day of transition. The transition from the type 2 to the type 3 SCFA profile was dependent on the increase in propionate concentration observed prior to cessation of breastfeeding, while the increase in butyrate and decrease in lactate and formate coincided with the cessation of breastfeeding. A relationship was also found between SCFAs and microbiota profiles, but these relationships were inconclusive. The effect of human milk on gut bacteria may be related to large amounts of lactoferrin (104), which limits the availability of iron ions necessary for bacteria to carry out enzymatic reactions and regulate gene expression (105). Bifidobacteria species are well adapted to conditions with low iron content (106), and lowering its availability results in decreased butyrate production (107, 108). Discontinuation of breastfeeding may result in a decrease in the amount of lactoferrin and an increase in the amount of free Fe, thus leading to increased production of butyrate in the intestines.

A comparison of SCFA fecal concentration between breastfeeding vs. formula feeding in age was presented in Table 5. Brink et al. observed that the levels of butyric acid in the feces of breastfed infants were higher than in formula-fed infants (36), which can prevent infections during this period of life (109). In children aged 3–5 months, it has been found (110) that exclusive breastfeeding was associated with lower absolute concentrations of total SCFAs, acetate, butyrate, propionate, valerate, isobutyrate, and isovalerate but higher concentrations of lactate. Moreover, the relative proportion of acetate was higher in the case of exclusive breastfeeding. This association was independent of the mode of delivery, intrapartum antibiotics administration, infant sex, age, site of enrolment, and maternal BMI or socioeconomic status.

**Table 5:** Comparison of SCFA fecal concentration between breastfeeding (B) vs. formula feeding (F) including data on analytical methods and formula composition.

**Reference**	**SCFAs analytical method**	**Microbiota analysis/** method	**Formula composition**	**Comparison of SCFA fecal concentration between B vs. formula feeding F during the study time**	**Comparison of SCFA feacl concentration between BF vs. formula FM in age**
Ben et al. (29)	GC	Culture	No	No	3 month: acetic acid: BF > FM.
Ben et al. (30)	GC	Culture	No	No	3, 6 month: acetic acid: BF > FM.
Berni Canani et al. (48)	GC	16SrRNA, V3-V4.	NA	NA	NA
Brink et al. (36)	UHPLC/ MS(Q-Exactive Hybrid Quadrupole-Orbitrap).	16SrRNA, V4.	No	No	3, 6 month: Butyric acid: BF > FM (also 9 month); Propionic acid: BF < FM; Isovaleric acid: BF > FM; Isobutyric acid: BF < FM.
Bazanella et al. (52)	UHPLC/ Q-ToF-MS.	16SrRNA,V3-V4/ strain identification - PCR.	Yes	Concentrations of propionic, butyric, isovaleric, and valeric acid increased over time in B and F group.	BF vs. FM: lower proportions of propionate, butyrate, valerate, isovalerate and higher pyruvic and lactic acid.
Bakker-Zierikzee et al. (31)	GC	FISH	Yes	No	5, 10 days; 4, 8, 12, 16 weeks: no difference of Acetate, Propionate, Butyrate, sum of isobutyrate, isovalerate and valerate SCFA between BF v FM.
Díaz et al. (37)	GC	16SrRNA, V3-V4.	NA	NA	NA
Differding et al. (38)	GC	16SrRNA, V4.	NA	NA	NA
Edwards et al. (32)	GLC	No	Yes	No	2, 4 weeks: Butyric acid, Propionic acid, Isovaleric acid: BF < FM; Acetic acid: BF = FM.
Fleming et. al. (53)	HPLC	16SrRNA	NA	NA	NA
Heath et al. (33)	GC	NA	NA	NA	NA
Holscher et al. (54)	GC/MS	FISH	Yes	No	0, 3, 6 weeks: acetate, propionate, and total SCFAs: BF < FM; isobutyrate BF > FM.
Iszatt et al. (39)	GC	16SrRNA, V4.	NA	NA	NA
Kien et al. (40)	GLC	NA	NA	NA	NA
Kim et al. (55)	1H-NMR spectroscopy	NA	NA	NA	NA
Knol et al. (56)	GC	FISH	Yes	NA	AN
Kok et al. (34)	GC	16SrRNA	Yes	0 → 30 day: propionate: increase of the concentration for amino acid formula; 0 → 60 day: butyrate and propionate: increase of the concentration for extensively hydrolyzed formula and amino acid formula. No changes in concentration for BF	30, 60 day: butyrate, propionate: FM (extensively hydrolyzed and amino acid formula) > BF; 60 day: total SCFA: F (amino acid formula) > BF.
Kosuwon, et al. (57)	GC	FISH	Yes	NA	NA
Liu et al. (58)	GC	PCR	Yes	No	3 weeks: Acetic acid FM < BF, Propionic acid and Butyric acid FM = BF.
Lund-Blix et al. (41)/ Dahl et al. (27)^a^	No	16SrRNA, V4.	NA	NA	NA
Maldonado et al. (59)	GC	Culture/PCR.	NA	NA	NA
Mentula et al. (60)	GC	Culture	NA	NA	NA
Midtvedt et al. (49)	GLC	NA	NA	NA	NA
Mohan et al. (61)	GC	Culture	NA	NA	NA
Nilsen et al. (42)	ND	16SrRNA, V4/ shotgun/ metaproteomics	NA	NA	NA
Nocerino et al. (62)	GC	16SrRNA	NA	NA	NA
Nogacka et al. (43)	GC	16SrRNA, V3.	NA	NA	NA
Oshiro et al. (63)	ND	reverse-transcription quantitative PCR	NA	NA	NA
Park et al. (44)	GC-MS	16SrRNA, V1-V3/ shotgun.	ND	ND	ND
Pourcyrous et al. (50)	IC	No		Preterm infants: 0 → 80 days: Butyrate: B: ↓, F: ↑; Propionate: B: ↑> F: ↑	Preterm inants: acetate, propionate, total SCFA: BF > FM
Quin et al. (35)	GC	16SrRNA, V3-V4.	NA	NA	NA
Rao et al. (45)	GC-MS	16SrRNA, V3-V4.	NA	NA	NA
Roduit et al. (46)	HPLC	No	NA	NA	NA
Sierra et al. (64)	GC	PCR	Yes	NA	NA
Stansbridge et al. (65)	GC	No	NA	NA	NA
Ta et al. (47)	LC/MS/MS	Metagenomic/ metatranscryptomic	NA	NA	NA
Tauchi et al. (51)	HPLC	16SrRNA, V1-V2.	NA	NA	NA
Underwood et al. (66)	HPLC	Culture/PCR	NA	NA	NA
Westerbeek et al. (67)	GC	FISH	NA	NA	NA
Wopereis et al. (69)	GC	16SrRNA, V3-V4	Yes	NA	8 week: acetate, propionate, butyrate, iso-valerate, iso-butyrate : BF = FM.
Wopereis et al. (68)	GC	16SrRNA, V3-V4	Yes	NA	4, 12, 26 weeks: proportions propionate, butyrate, isobutyrate and isovalerate BF < FM.

*GC, gas chromatography; UHPLC, ultra-high performance liquid chromatography; MS, mass spectrometry; HPLC, high performance liquid chromatography; Q-ToF, quadrupole time of flight; 1H-NMR, proton nuclear magnetic resonance; FISH, fluorescence in situ hybridization; PCR, polymerase chain reaction; GLC, gas liquid chromatography; IC, ion chromatography; LC, liquid chromatography; ND, no data; NA, not applicable.*

a *source of data indicated by Lund-Blix et al. (41)*.

Edwards et al. also found an elevation in the relative proportions of acetate (76%) in breastfed infants at 4 weeks of age (32). The higher relative acetate abundance in exclusively breastfed infants may be partly due to the presence of HMO in breast milk that is not present in the infant formula. HMOs, the third largest component of human milk, are soluble carbohydrates that are not digested by the host and are substrates for selected gut microbiota (111). There is a close relationship between the infant's gut microbiota, that of the mother's milk, and the composition of human milk oligosaccharides (HMO) (112–115). Recent studies have shown that human milk microbiota can directly colonize an infant's intestines, and the effect of human milk on the infant's intestinal microbiota is dose-dependent (8). The microbiota in breast milk changes during lactation and is different in exclusively breastfeeding and non-breastfeeding mothers (116, 117). The abundance of gut microbiota in breastfed infants, especially bifidobacteria species, is correlated with maternal HMO content and its catabolic activity (82, 118, 119). Bifidobacterium species are a narrow group of gut bacteria capable of metabolizing HMO (120) and are therefore over-represented in the microbiota of breastfed infants compared to formula-fed babies (1, 93, 121). The results of studies by Azad et al. showed that the *Bifidobacteriaceae* family is enriched in the case of breastfeeding (93).

*Bifidobacterium* has been shown to metabolize HMO to produce acetate and lactate (122, 123). Martin et al. observed that the increase in the number of bifidobacteria corresponds to the increase in the acetate concentration in the stool (124). Although evidence is limited, higher acetate levels in breastfed infants may protect against gut pathogens and allergic diseases (125). Fukudo et al. showed that acetate, produced by bifidobacteria, improved intestinal defenses and protected against *Escherichia coli* O157: H7 in mice (122). Thorburn et al. (126) proposed that the inhibition of HDAC by acetate, shown in an adult mouse model, increases the transcription of the Foxp3 gene, which may promote Treg- suppression of airway inflammation and induction of oral tolerance. In infants, Arrieta et al. found that a decreased amount of acetate in the stool at 3 months of age was associated with allergic disease in later infancy (127).

Other components of breastmilk can also affect gut microbiota development (128, 129). For instance, maternally derived antibodies: IgA, IgM, IgG and secretory IgM (SIgM) and IgA (SIgA) have the ability to bind the microbes and consequently protect against respiratory and gastrointestinal infections (130). Cytokines and growth factors are mother-to-child signals synthesized to improve action of leukocytes. What is more, defensins and cathelicidins that breast milk contains are well-known antimicrobial molecules (131). Paralelly, breast-milk originated lysozyme breaks the microbial cell walls *via* lysis process. Free amino acids which are present in breast milk are essential for growth of the nervous tissue, eye and intestines and protein breaking enzymes support the hydrolysis of the milk proteins (132). Breast milk also stimulates the secretion of calprotectin and zonulin, both involved in gut barrier and microbiota development (133).

We have also observed lower absolute concentrations of SCFAs in exclusively breastfed infants, which is in line with other published studies. In 111 stool samples analyzed by NMR, Martin et al. found lower concentrations of SCFAs at 3 and 6 months of age in breastfed infants born to overweight or obese mothers (134). In a small study of 4 infants using GC and LC mass spectrometry, valerate and isovalerate concentrations were higher in formula-fed infants, the latter more than 40 times higher than in breastfed infants (135). The higher absolute concentrations of SCFAs observed in formula-fed infants may result from the greater bacterial diversity observed in these infants compared to purely breastfed infants (93) and thus a greater ability to metabolize substrates present in the intestines. The exclusivity of breastfeeding was inversely related to both the richness and diversity of the microbiota (93). The observed differences may also result from differences in the composition or absorption of breast milk compared to the modified milk and thus from differences in the availability of substrates. In addition, higher concentrations of branched-chain fatty acids, valerate, isobutyrate, and isovalerate, derived from amino acid metabolism, indicate reduced protein absorption or excessive protein intake [potentially due to the higher protein content of the formula compared to human milk (136) in formula-fed babies]. The availability of these substrates is likely to increase the abundance of proteolytic bacteria, such as *Bacteroides* and *Clostridia*, seen in formula-fed infants (93). Higher concentrations of proteolytic metabolites in mineral formula-fed infants may also be due to the reduced availability of carbohydrates without HMO and, thus, greater energy extraction from protein metabolism. Chow et al. (135) showed that in the absence of fermentable carbohydrates in the feces of both breast- and formula-fed infants, mainly metabolites were produced, indicating fermentation of proteins; their production was reduced with the addition of various fermentable HMO-like carbohydrate substrates. Increased fecal SCFAs in formula-fed infants may have metabolic consequences. Several studies have reported higher fecal SCFAs in adults and overweight children compared to their lean counterparts (137–141) and correlations with other metabolic risk factors (141). While causality has not yet been established, the authors of these studies hypothesize that a higher concentration of SCFAs may reflect the increased ability of the gut microbiota to obtain energy from the diet.

Our meta-analysis also had some limitations. We observed significant heterogeneity in the papers analyzed due to different analytical methods. For the same reason, we did not compare the results obtained to the gut microbiota composition. In addition, the authors of the papers did not often provide the composition of the dietary mixtures. All this information is included in Table 5. Another limitation of our study was the indirect measurement of luminal metabolites by analyzing stool samples. The concentration of metabolites in the feces is a function of production, absorption, use by other microorganisms, and stool transit time. Few studies have examined whether the content of SCFA in stool is a reliable indicator of luminal content, especially in early childhood, although it is estimated that 95% of SCFA produced in the gut is rapidly absorbed, and only 5% is excreted in the feces (15). In a study of healthy adults, Vogt and Wolever found that fecal acetate concentrations are inversely correlated with acetate absorption, suggesting that fecal acetate concentrations may reflect intestinal absorption rather than production (142). However, given that the potential of fecal metabolite analysis is to provide a biomarker for predicting future disease risk, analysis of stool samples provides a non-invasive and cost-effective method for epidemiological cohort studies. Our sampling was also only at a one-time point in infancy; therefore, our data do not provide comprehensive information on trends in metabolites over time concerning diet. A limitation of our work is the inability to assess the long-term health consequences of SCFAs stool concentration.

We can conclude that the feeding mode influences the content of SCFAs in stools in early life, with artificial feeding being associated with a higher content of SCFAs in stools and breastfeeding with the occurrence of more significant differences in the concentration of SCFAs in stools between different periods of a child's early life. There is a need for long-term assessment of the impact of observed differences on health in later life and unification of analytical methods and methodologies for SCFAs testing in young children.

## Data Availability Statement

The original contributions presented in the study are included in the article/Supplementary Material, further inquiries can be directed to the corresponding author.

## Author Contributions

IŁ and BŁ: conceptualization. KS-Ż: methods and statistical analysis. LS, MF-T, and PT: investigation. IŁ: original draft. All authors: review and editing. All authors contributed to the article and approved the submitted version.

## Conflict of Interest

The authors declare that the research was conducted in the absence of any commercial or financial relationships that could be construed as a potential conflict of interest.

## Publisher's Note

All claims expressed in this article are solely those of the authors and do not necessarily represent those of their affiliated organizations, or those of the publisher, the editors and the reviewers. Any product that may be evaluated in this article, or claim that may be made by its manufacturer, is not guaranteed or endorsed by the publisher.

## References

[1] Bäckhed F Roswall J Peng Y Feng Q Jia H Kovatcheva-Datchary P . Dynamics and stabilization of the human gut microbiome during the first year of life. Cell Host Microbe. (2015) 17:690–703. 10.1016/j.chom.2015.04.004 25974306

[2] Stewart CJ Steward C Ajami NJ O'Brien JL ORein J Hutchinson DS . Temporal development of the gut microbiome in early childhood from the TEDDY study. Nature. (2018) 562:583–8. 10.1038/s41586-018-0617-x 30356187 PMC6415775

[3] Mesa MD Loureiro B Iglesia I Fernandez Gonzalez S Llurba Olivé E García Algar O . The evolving microbiome from pregnancy to early infancy: a comprehensive review. Nutrients. (2020) 12:133. 10.3390/nu12010133 PMC7019214 31906588

[4] Forbes JD Azad MB Vehling L Tun HM Konya TB Guttman DS . Association of exposure to formula in the hospital and subsequent infant feeding practices with gut microbiota and risk of overweight in the first year of life. JAMA Pediatr. (2018) 172:e181161. 10.1001/jamapediatrics.2018.1161 29868719 PMC6137517

[5] Baumann-Dudenhoeffer AM D'Souza AW Tarr PI Warner BB Dantas G. Infant diet and maternal gestational weight gain predict early metabolic maturation of gut microbiomes. Nat Med. (2018) 24:1822–9. 10.1038/s41591-018-0216-2 30374198 PMC6294307

[6] De Leoz MLA Kalanetra KM Bokulich NA Strum JS Underwood MA German JB . Human milk glycomics and gut microbial genomics in infant feces show a correlation between human milk oligosaccharides and gut microbiota: a proof-of-concept study. J Proteome Res. (2015) 14:491–502. 10.1021/pr500759e 25300177 PMC4286166

[7] Ho NT Li F Lee-Sarwar KA Tun HM Brown BP Pannaraj PS . Meta-analysis of effects of exclusive breastfeeding on infant gut microbiota across populations. Nat Commun. (2018) 9:4169. 10.1038/s41467-018-06473-x 30301893 PMC6177445

[8] Pannaraj PS Li F Cerini C Bender JM Yang S Rollie A . Association between breast milk bacterial communities and establishment and development of the infant gut microbiome. JAMA Pediatr. (2017) 171:647–54. 10.1001/jamapediatrics.2017.0378 28492938 PMC5710346

[9] Jakobsson HE Abrahamsson TR Jenmalm MC Harris K Quince C Jernberg C . Decreased gut microbiota diversity, delayed Bacteroidetes colonisation and reduced Th1 responses in infants delivered by caesarean section. Gut. (2014) 63:559–66. 10.1136/gutjnl-2012-303249 23926244

[10] Korpela K Salonen A Saxen H Nikkonen A Peltola V Jaakkola T . Antibiotics in early life associate with specific gut microbiota signatures in a prospective longitudinal infant cohort. Pediatr Res. (2020) 88:438–43. 10.1038/s41390-020-0761-5 31954376

[11] Soderborg TK Clark SE Mulligan CE Janssen RC Babcock L Ir D . The gut microbiota in infants of obese mothers increases inflammation and susceptibility to NAFLD. Nat Commun. (2018) 9:4462. 10.1038/s41467-018-06929-0 30367045 PMC6203757

[12] Lazar V Ditu LM Pircalabioru GG Gheorghe I Curutiu C Holban AM . Aspects of gut microbiota and immune system interactions in infectious diseases, immunopathology, and cancer. Front Immunol. (2018) 9:1830. 10.3389/fimmu.2018.01830 30158926 PMC6104162

[13] Olin A Henckel E Chen Y Lakshmikanth T Pou C Mikes J . stereotypic immune system development in newborn children. Cell. (2018) 174:1277–92.e14. 10.1016/j.cell.2018.06.045 30142345 PMC6108833

[14] Tun HM Bridgman SL Chari R Field CJ Guttman DS Becker AB . Roles of birth mode and infant gut microbiota in intergenerational transmission of overweight and obesity from mother to offspring. JAMA Pediatr. (2018) 172:368–77. 10.1001/jamapediatrics.2017.5535 29459942 PMC5875322

[15] den Besten G van Eunen K Groen AK Venema K Reijngoud DJ Bakker BM. The role of short-chain fatty acids in the interplay between diet, gut microbiota, and host energy metabolism. J Lipid Res. (2013) 54:2325–40. 10.1194/jlr.R036012 23821742 PMC3735932

[16] Roy CC Kien CL Bouthillier L Levy E. Short-chain fatty acids: ready for prime time? Nutr Clin Pract. (2006) 21:351–66. 10.1177/0115426506021004351 16870803

[17] van Hoek MJA Merks RMH. Redox balance is key to explaining full vs. partial switching to low-yield metabolism. BMC Syst Biol. (2012) 6:22. 10.1186/1752-0509-6-22 22443685 PMC3384451

[18] von Martels JZH Sadaghian Sadabad M Bourgonje AR Blokzijl T Dijkstra G Faber KN . The role of gut microbiota in health and disease: *in vitro* modeling of host-microbe interactions at the aerobe-anaerobe interphase of the human gut. Anaerobe. (2017) 44:3–12. 10.1016/j.anaerobe.2017.01.001 28062270

[19] Rowland I Gibson G Heinken A Scott K Swann J Thiele I . Gut microbiota functions: metabolism of nutrients and other food components. Eur J Nutr. (2018) 57:1–24. 10.1007/s00394-017-1445-8 PMC5847071 28393285

[20] Steliou K Boosalis MS Perrine SP Sangerman J Faller DV. Butyrate histone deacetylase inhibitors. Biores Open Access. (2012) 1:192–8. 10.1089/biores.2012.0223 23514803 PMC3559235

[21] Frost G Sleeth ML Sahuri-Arisoylu M Lizarbe B Cerdan S Brody L . The short-chain fatty acid acetate reduces appetite via a central homeostatic mechanism. Nat Commun. (2014) 5:3611. 10.1038/ncomms4611 24781306 PMC4015327

[22] Lawhon SD Maurer R Suyemoto M Altier C. Intestinal short-chain fatty acids alter *Salmonella typhimurium* invasion gene expression and virulence through BarA/SirA. Mol Microbiol. (2002) 46:1451–64. 10.1046/j.1365-2958.2002.03268.x 12453229

[23] Nakanishi N Tashiro K Kuhara S Hayashi T Sugimoto N Tobe T. Regulation of virulence by butyrate sensing in enterohaemorrhagic *Escherichia coli*. Microbiology. (2009) 155:521–30. 10.1099/mic.0.023499-0 19202100

[24] DerSimonian R Laird N. Meta-analysis in clinical trials. Control Clin Trials. (1986) 7:177–88. 10.1016/0197-2456(86)90046-2 3802833

[25] Egger M Davey Smith G Schneider M Minder C. Bias in meta-analysis detected by a simple, graphical test. BMJ. (1997) 315:629–34. 10.1136/bmj.315.7109.629 9310563 PMC2127453

[26] Duval S Tweedie R. A nonparametric “trim and fill” method of accounting for publication bias in meta-analysis. J Am Stat Assoc. (2000) 95:89–98. 10.1080/01621459.2000.10473905

[27] Dahl C Stigum H Valeur J Iszatt N Lenters V Peddada S . Preterm infants have distinct microbiomes not explained by mode of delivery, breastfeeding duration or antibiotic exposure. Int J Epidemiol. (2018) 47:1658–69. 10.1093/ije/dyy064 29688458

[28] Westerbeek EA van den Berg JP Lafeber HN Fetter WP Boehm G Twisk JW . Neutral and acidic oligosaccharides in preterm infants: a randomized, double-blind, placebo-controlled trial. Am J Clin Nutr. (2010) 91:679–86. 10.3945/ajcn.2009.28625 20032496

[29] Ben XM Li J Feng ZT Shi SY Lu YD Chen R . Low level of galacto-oligosaccharide in infant formula stimulates growth of intestinal Bifidobacteria and Lactobacilli. World J Gastroenterol. (2008) 14:6564–8. 10.3748/wjg.14.6564 19030213 PMC2773347

[30] Ben XM Zhou XY Zhao WH Yu WL Pan W Zhang WL . Supplementation of milk formula with galacto-oligosaccharides improves intestinal micro-flora and fermentation in term infants. Chin Med J. (2004) 117:927–31. 15198901

[31] Bakker-Zierikzee AM Alles MS Knol J Kok FJ Tolboom JJM Bindels JG. Effects of infant formula containing a mixture of galacto- and fructo-oligosaccharides or viable *Bifidobacterium animalis* on the intestinal microflora during the first 4 months of life. Br J Nutr. (2005) 94:783–90. 10.1079/BJN20051451 16277782

[32] Edwards CA Parrett AM Balmer SE Wharton BA. Faecal short chain fatty acids in breast-fed and formula-fed babies. Acta Paediatr. (1994) 83:459–62. 10.1111/j.1651-2227.1994.tb13059.x 8086719

[33] Heath ALM Haszard JJ Galland BC Lawley B Rehrer NJ Drummond LN . Association between the faecal short-chain fatty acid propionate and infant sleep. Eur J Clin Nutr. (2020) 74:1362–5. 10.1038/s41430-019-0556-0 31969698

[34] Kok CR Brabec B Chichlowski M Harris CL Moore N Wampler JL . Stool microbiome, pH and short/branched chain fatty acids in infants receiving extensively hydrolyzed formula, amino acid formula, or human milk through two months of age. BMC Microbiol. (2020) 20:337. 10.1186/s12866-020-01991-5 33167908 PMC7650147

[35] Quin C Estaki M Vollman DM Barnett JA Gill SK Gibson DL. Probiotic supplementation and associated infant gut microbiome and health: a cautionary retrospective clinical comparison. Sci Rep. (2018) 8:8283. 10.1038/s41598-018-26423-3 29844409 PMC5974413

[36] Brink LR Mercer KE Piccolo BD Chintapalli SV Elolimy A Bowlin AK . Neonatal diet alters fecal microbiota and metabolome profiles at different ages in infants fed breast milk or formula. Am J Clin Nutr. (2020) 111:1190–202. 10.1093/ajcn/nqaa076 32330237 PMC7266684

[37] Díaz M Guadamuro L Espinosa-Martos I Mancabelli L Jiménez S Molinos-Norniella C . Microbiota and derived parameters in fecal samples of infants with non-IgE Cow's milk protein allergy under a restricted diet. Nutrients. (2018) 10:1481. 10.3390/nu10101481 PMC6213916 30314304

[38] Differding MK Benjamin-Neelon SE Hoyo C Østbye T Mueller NT. Timing of complementary feeding is associated with gut microbiota diversity and composition and short chain fatty acid concentrations over the first year of life. BMC Microbiology. (2020) 20:56. 10.1186/s12866-020-01723-9 32160858 PMC7065329

[39] Iszatt N. Environmental toxicants in breast milk of Norwegian mothers and gut bacteria composition and metabolites in their infants at one month. Nor Epidemiol. (2019) 28:86. 10.1186/s40168-019-0645-2 PMC6393990 30813950

[40] Kien CL Liechty EA Mullett MD. Contribution of low-molecular-weight compounds to the fecal excretion of carbohydrate energy in premature infants. Gastroenterology. (1990) 99:165–74. 10.1016/0016-5085(90)91244-Z 2344923

[41] Lund-Blix NA Tapia G Mårild K Brantsæter AL Eggesbø M Mandal S . Maternal fibre and gluten intake during pregnancy and risk of childhood celiac disease: the MoBa study. Sci Rep. (2020) 10:16439. 10.1038/s41598-020-73244-4 33009438 PMC7532434

[42] Nilsen M Madelen Saunders C Leena Angell I Arntzen MØ Lødrup Carlsen KC Carlsen KH . Butyrate levels in the transition from an infant- to an adult-like gut microbiota correlate with bacterial networks associated with *Eubacterium rectale* and *Ruminococcus gnavus*. Genes. (2020) 11:1245. 10.3390/genes11111245 PMC7690385 33105702

[43] Nogacka A Salazar N Suárez M Milani C Arboleya S Solís G . Impact of intrapartum antimicrobial prophylaxis upon the intestinal microbiota and the prevalence of antibiotic resistance genes in vaginally delivered full-term neonates. Microbiome. (2017) 5:93. 10.1186/s40168-017-0313-3 28789705 PMC5549288

[44] Park YM Lee SY Kang MJ Kim BS Lee MJ Jung SS . Imbalance of gut streptococcus, clostridium, and *Akkermansia* determines the natural course of atopic dermatitis in infant. Allergy Asthma Immunol Res. (2020) 12:322–37. 10.4168/aair.2020.12.2.322 32009325 PMC6997289

[45] Rao SC Esvaran M Patole SK Simmer KN Gollow I Keil A . Gut microbiota in neonates with congenital gastrointestinal surgical conditions: a prospective study. Pediatr Res. (2020) 88:878–86. 10.1038/s41390-020-0824-7 32179871 PMC7223116

[46] Roduit C Frei R Ferstl R Loeliger S Westermann P Rhyner C . High levels of butyrate and propionate in early life are associated with protection against atopy. Allergy. (2019) 74:799–809. 10.1111/all.13660 30390309

[47] Ta LDH Chan JCY Yap GC Purbojati RW Drautz-Moses DI Koh YM . A compromised developmental trajectory of the infant gut microbiome and metabolome in atopic eczema. Gut Microbes. (2020) 12:1801964. 10.1080/19490976.2020.1801964 PMC7553750 33023370

[48] Berni Canani R De Filippis F Nocerino R Paparo L Di Scala C Cosenza L . Gut microbiota composition and butyrate production in children affected by non-IgE-mediated cow's milk allergy. Sci Rep. (2018) 8:12500. 10.1038/s41598-018-30428-3 30131575 PMC6104073

[49] Midtvedt AC Carlstedt-Duke B Norin KE Saxerholt H Midtvedt T. Development of five metabolic activities associated with the intestinal microflora of healthy infants. J Pediatr Gastroenterol Nutr. (1988) 7:559–67. 10.1097/00005176-198807000-00014 3135382

[50] Pourcyrous M Nolan VG Goodwin A Davis SL Buddington RK. Fecal short-chain fatty acids of very-low-birth-weight preterm infants fed expressed breast milk or formula. J Pediatr Gastroenterol Nutr. (2014) 59:725–31. 10.1097/MPG.0000000000000515 25079478

[51] Tauchi H Yahagi K Yamauchi T Hara T Yamaoka R Tsukuda N . Gut microbiota development of preterm infants hospitalised in intensive care units. Benef Microbes. (2019) 10:641–51. 10.3920/BM2019.0003 31179713

[52] Bazanella M Maier TV Clavel T Lagkouvardos I Lucio M Maldonado-Gòmez MX . Randomized controlled trial on the impact of early-life intervention with bifidobacteria on the healthy infant fecal microbiota and metabolome. Am J Clin Nutr. (2017) 106:1274–86. 10.3945/ajcn.117.157529 28877893

[53] Fleming P Wilks M Eaton S Panton N Hutchinson R Akyempon A . Bifidobacterium breve BBG-001 and intestinal barrier function in preterm babies: exploratory studies from the PiPS trial. Pediatr Res. (2020) 89:1818–24. 10.1038/s41390-020-01135-5 32947603

[54] Holscher HD Faust KL Czerkies LA Litov R Ziegler EE Lessin H . Effects of prebiotic-containing infant formula on gastrointestinal tolerance and fecal microbiota in a randomized controlled trial. J Parenter Enteral Nutr. (2012) 36 (1 Suppl.):95S−105S. 10.1177/0148607111430087 22237884

[55] Kim HK Rutten NBMM Besseling-van der Vaart I Niers LEM Choi YH Rijkers GT . Probiotic supplementation influences faecal short chain fatty acids in infants at high risk for eczema. Benef Microbes. (2015) 6:783–90. 10.3920/BM2015.0056 26565082

[56] Knol J Scholtens P Kafka C Steenbakkers J Gro S Helm K . Colon Microflora in infants fed formula with galacto- and fructo-oligosaccharides: more like breast-fed infants. J Pediatr Gastroenterol Nutr. (2005) 40:36–42. 10.1097/00005176-200501000-00007 15625424

[57] Kosuwon P Lao-Araya M Uthaisangsook S Lay C Bindels J Knol J . A synbiotic mixture of scGOS/lcFOS and *Bifidobacterium breve* M-16V increases faecal Bifidobacterium in healthy young children. Benef Mirbobes. (2018) 9:541–52. 10.3920/BM2017.0110 29633642

[58] Liu Z Roy NC Guo Y Jia H Ryan L Samuelsson L . Human breast milk and infant formulas differentially modify the intestinal microbiota in human infants and host physiology in rats. J Nutr. (2016) 146:191–9. 10.3945/jn.115.223552 26674765

[59] Maldonado J Lara-Villoslada F Sierra S Sempere L Gómez M Rodriguez JM . Safety and tolerance of the human milk probiotic strain *Lactobacillus salivarius* CECT5713 in 6-month-old children. Nutrition. (2010) 26:1082–7. 10.1016/j.nut.2009.08.023 20018483

[60] Mentula S Tuure T Koskenala R Korpela R Könönen E. Microbial composition and fecal fermentation end products from colicky infants – a probiotic supplementation pilot. Microb Ecol Health Dis. (2008) 20:37–47. 10.1080/08910600801933846

[61] Mohan R Koebnick C Schildt J Mueller M Radke M Blaut M. Effects of *Bifidobacterium lactis* Bb12 supplementation on body weight, fecal pH, acetate, lactate, calprotectin, and IgA in preterm infants. Pediatr Res. (2008) 64:418–22. 10.1203/PDR.0b013e318181b7fa 18552710

[62] Nocerino R Filippis FD Cecere G Marino A Micillo M Scala CD . The therapeutic efficacy of *Bifidobacterium animalis* subsp. lactis BB-12® in infant colic: A randomized, double blind, placebo-controlled trial. Aliment Pharmacol Ther. (2020) 51:110–20. 10.1111/apt.15561 31797399 PMC6973258

[63] Oshiro T Nagata S Wang C Takahashi T Tsuji H Asahara T . Bifidobacterium supplementation of colostrum and breast milk enhances weight gain and metabolic responses associated with microbiota establishment in very-preterm infants. BMH. (2019) 4:1–10. 10.1159/000502935 PMC6985890 31993433

[64] Sierra C Bernal MJ Blasco J Martínez R Dalmau J Ortuño I . Prebiotic effect during the first year of life in healthy infants fed formula containing GOS as the only prebiotic: a multicentre, randomized, double-blind and placebo-controlled trial. Eur J Nutr. (2015) 54:89–99. 10.1007/s00394-014-0689-9 24671237 PMC4303717

[65] Stansbridge EM Walker V Hall MA Smith SL Millar MR Bacon C . Effects of feeding premature infants with Lactobacillus GG on gut fermentation. Arch Dis Child. (1993) 69:488–92. 10.1136/adc.69.5_Spec_No.488 8285751 PMC1029590

[66] Underwood MA Salzman NH Bennett SH Barman M Mills DA Marcobal A . A randomized placebo-controlled comparison of 2 prebiotic/probiotic combinations in preterm infants: impact on weight gain, intestinal microbiota, and fecal short-chain fatty acids. J Pediatr Gastroenterol Nutr. (2009) 48:216–25. 10.1097/MPG.0b013e31818de195 19179885 PMC2743418

[67] Westerbeek E a. M, Slump RA, Lafeber HN, Knol J, Georgi G, Fetter WPF, et al. The effect of enteral supplementation of specific neutral and acidic oligosaccharides on the faecal microbiota and intestinal microenvironment in preterm infants. Eur J Clin Microbiol Infect Dis. (2013) 32:269–76. 10.1007/s10096-012-1739-y 22961006

[68] Wopereis H Sim K Shaw A Warner JO Knol J Kroll JS. Intestinal microbiota in infants at high risk for allergy: effects of prebiotics and role in eczema development. J Allergy Clin Immunol. (2018) 141:1334–42.e5. 10.1016/j.jaci.2017.05.054 28866384

[69] Wopereis H van Ampting MTJ Cetinyurek-Yavuz A Slump R Candy DCA Butt AM . A specific synbiotic-containing amino acid-based formula restores gut microbiota in non-IgE mediated cow's milk allergic infants: a randomized controlled trial. Clin Trans Allergy. (2019) 9:27. 10.1186/s13601-019-0267-6 PMC6543596 31164972

[70] Cummings JH. Short chain fatty acids in the human colon. Gut. (1981) 22:763–79. 10.1136/gut.22.9.763 7028579 PMC1419865

[71] Koenig JE Spor A Scalfone N Fricker AD Stombaugh J Knight R . Succession of microbial consortia in the developing infant gut microbiome. Proc Natl Acad Sci USA. (2011) 108 (Suppl. 1):4578–85. 10.1073/pnas.1000081107 20668239 PMC3063592

[72] Sghir A Gramet G Suau A Rochet V Pochart P Dore J. Quantification of bacterial groups within human fecal flora by oligonucleotide probe hybridization. Appl Environ Microbiol. (2000) 66:2263–6. 10.1128/AEM.66.5.2263-2266.2000 10788414 PMC101487

[73] Cummings JH Macfarlane GT. The control and consequences of bacterial fermentation in the human colon. J Appl Bacteriol. (1991) 70:443–59. 10.1111/j.1365-2672.1991.tb02739.x 1938669

[74] Lopetuso LR Scaldaferri F Petito V Gasbarrini A. Commensal Clostridia: leading players in the maintenance of gut homeostasis. Gut Pathog. (2013) 5:23. 10.1186/1757-4749-5-23 23941657 PMC3751348

[75] Louis P Flint HJ. Diversity, metabolism and microbial ecology of butyrate-producing bacteria from the human large intestine. FEMS Microbiol Lett. (2009) 294:1–8. 10.1111/j.1574-6968.2009.01514.x 19222573

[76] Hiippala K Jouhten H Ronkainen A Hartikainen A Kainulainen V Jalanka J . The potential of gut commensals in reinforcing intestinal barrier function and alleviating inflammation. Nutrients. (2018) 10:E988. 10.3390/nu10080988 30060606 PMC6116138

[77] Turroni F Milani C Duranti S Mahony J van Sinderen D Ventura M. Glycan utilization and cross-feeding activities by bifidobacteria. Trends Microbiol. (2018) 26:339–50. 10.1016/j.tim.2017.10.001 29089173

[78] Bunesova V Lacroix C Schwab C. Mucin cross-feeding of infant bifidobacteria and *Eubacterium hallii*. Microb Ecol. (2018) 75:228–38. 10.1007/s00248-017-1037-4 28721502

[79] Derrien M Vaughan EE Plugge CM de Vos WM. *Akkermansia muciniphila* gen. nov., sp. nov., a human intestinal mucin-degrading bacterium. Int J Syst Evol Microbiol. (2004) 54:1469–76. 10.1099/ijs.0.02873-0 15388697

[80] Tamburini S Shen N Wu HC Clemente JC. The microbiome in early life: implications for health outcomes. Nat Med. (2016) 22:713–22. 10.1038/nm.4142 27387886

[81] Nagata R Nagano H Ogishima D Nakamura Y Hiruma M Sugita T. Transmission of the major skin microbiota, Malassezia, from mother to neonate. Pediatr Int. (2012) 54:350–5. 10.1111/j.1442-200X.2012.03563.x 22300401

[82] Bender JM Li F Martelly S Byrt E Rouzier V Leo M . Maternal HIV infection influences the microbiome of HIV uninfected infants. Sci Transl Med. (2016) 8:349ra100. 10.1126/scitranslmed.aaf5103 PMC5301310 27464748

[83] Schanche M Avershina E Dotterud C Øien T Storrø O Johnsen R . High-resolution analyses of overlap in the microbiota between mothers and their children. Curr Microbiol. (2015) 71:283–90. 10.1007/s00284-015-0843-5 26044992

[84] Azad MB Konya T Maughan H Guttman DS Field CJ Chari RS . Gut microbiota of healthy Canadian infants: profiles by mode of delivery and infant diet at 4 months. CMAJ. (2013) 185:385–94. 10.1503/cmaj.121189 23401405 PMC3602254

[85] Gomez-Llorente C Plaza-Diaz J Aguilera M Muñoz-Quezada S Bermudez-Brito M Peso-Echarri P . Three main factors define changes in fecal microbiota associated with feeding modality in infants. J Pediatr Gastroenterol Nutr. (2013) 57:461–6. 10.1097/MPG.0b013e31829d519a 23752082

[86] Gregory KE Samuel BS Houghteling P Shan G Ausubel FM Sadreyev RI . Influence of maternal breast milk ingestion on acquisition of the intestinal microbiome in preterm infants. Microbiome. (2016) 4:68. 10.1186/s40168-016-0214-x 28034306 PMC5200970

[87] Sordillo JE Zhou Y McGeachie MJ Ziniti J Lange N Laranjo N . Factors influencing the infant gut microbiome at age 3-6 months: findings from the ethnically diverse vitamin D antenatal asthma reduction trial (VDAART). J Allergy Clin Immunol. (2017) 139:482–91.e14. 10.1016/j.jaci.2016.08.045 27746239 PMC5303123

[88] Timmerman HM Rutten NBMM Boekhorst J Saulnier DM Kortman GAM Contractor N . Intestinal colonisation patterns in breastfed and formula-fed infants during the first 12 weeks of life reveal sequential microbiota signatures. Sci Rep. (2017) 7:8327. 10.1038/s41598-017-08268-4 28827640 PMC5567133

[89] Bezirtzoglou E Tsiotsias A Welling GW. Microbiota profile in feces of breast- and formula-fed newborns by using fluorescence in situ hybridization (FISH). Anaerobe. (2011) 17:478–82. 10.1016/j.anaerobe.2011.03.009 21497661

[90] Davis MY Zhang H Brannan LE Carman RJ Boone JH. Rapid change of fecal microbiome and disappearance of *Clostridium difficile* in a colonized infant after transition from breast milk to cow milk. Microbiome. (2016) 4:53. 10.1186/s40168-016-0198-6 27717398 PMC5055705

[91] Wood LF Brown BP Lennard K Karaoz U Havyarimana E Passmore JAS . Feeding-related gut microbial composition associates with peripheral T-cell activation and mucosal gene expression in African infants. Clin Infect Dis. (2018) 67:1237–46. 10.1093/cid/ciy265 29659737 PMC6455922

[92] Thompson AL Monteagudo-Mera A Cadenas MB Lampl ML Azcarate-Peril MA. Milk- and solid-feeding practices and daycare attendance are associated with differences in bacterial diversity, predominant communities, and metabolic and immune function of the infant gut microbiome. Front Cell Infect Microbiol. (2015) 5:3. 10.3389/fcimb.2015.00003 25705611 PMC4318912

[93] Azad MB Konya T Persaud RR Guttman DS Chari RS Field CJ . Impact of maternal intrapartum antibiotics, method of birth and breastfeeding on gut microbiota during the first year of life: a prospective cohort study. BJOG. (2016) 123:983–93. 10.1111/1471-0528.13601 26412384

[94] Hesla HM Stenius F Jäderlund L Nelson R Engstrand L Alm J . Impact of lifestyle on the gut microbiota of healthy infants and their mothers—the ALADDIN birth cohort. FEMS Microbiol Ecol. (2014) 90:791–801. 10.1111/1574-6941.12434 25290507

[95] Lagier JC Million M Hugon P Armougom F Raoult D. Human gut microbiota: repertoire and variations. Front Cell Infect Microbiol. (2012) 2:136. 10.3389/fcimb.2012.00136 23130351 PMC3487222

[96] Schwiertz A Le Blay G Blaut M. Quantification of different Eubacterium spp. in human fecal samples with species-specific 16S rRNA-targeted oligonucleotide probes. Appl Environ Microbiol. (2000) 66:375–82. 10.1128/AEM.66.1.375-382.2000 10618251 PMC91833

[97] Vael C Verhulst SL Nelen V Goossens H Desager KN. Intestinal microflora and body mass index during the first three years of life: an observational study. Gut Pathog. (2011) 3:8. 10.1186/1757-4749-3-8 21605455 PMC3118227

[98] Brook I. Veillonella infections in children. J Clin Microbiol. (1996) 34:1283–5. 10.1128/jcm.34.5.1283-1285.1996 8727920 PMC228999

[99] Stuebe A. The risks of not breastfeeding for mothers and infants. Rev Obstet Gynecol. (2009) 2:222–31. 10.3909/riog0093 20111658 PMC2812877

[100] Yan J Liu L Zhu Y Huang G Wang PP. The association between breastfeeding and childhood obesity: a meta-analysis. BMC Public Health. (2014) 14:1267. 10.1186/1471-2458-14-1267 25495402 PMC4301835

[101] Cardwell CR Stene LC Ludvigsson J Rosenbauer J Cinek O Svensson J . Breast-feeding and childhood-onset type 1 diabetes: a pooled analysis of individual participant data from 43 observational studies. Diabetes Care. (2012) 35:2215–25. 10.2337/dc12-0438 22837371 PMC3476923

[102] Tsukuda N Yahagi K Hara T Watanabe Y Matsumoto H Mori H . Key bacterial taxa and metabolic pathways affecting gut short-chain fatty acid profiles in early life. ISME J. (2021) 15:2574–90. 10.1038/s41396-021-00937-7 33723382 PMC8397723

[103] de Muinck EJ Trosvik P. Individuality and convergence of the infant gut microbiota during the first year of life. Nat Commun. (2018) 9:2233. 10.1038/s41467-018-04641-7 29884786 PMC5993781

[104] Czosnykowska-Łukacka M Orczyk-Pawiłowicz M Broers B Królak-Olejnik B. Lactoferrin in human milk of prolonged lactation. Nutrients. (2019) 11:E2350. 10.3390/nu11102350 31581741 PMC6835443

[105] Page MGP. The role of iron and siderophores in infection, and the development of siderophore antibiotics. Clin Infect Dis. (2019) 69 (Suppl_7):S529–37. 10.1093/cid/ciz825 31724044 PMC6853763

[106] Vazquez-Gutierrez P Stevens MJA Gehrig P Barkow-Oesterreicher S Lacroix C Chassard C. The extracellular proteome of two Bifidobacterium species reveals different adaptation strategies to low iron conditions. BMC Genomics. (2017) 18:41. 10.1186/s12864-016-3472-x 28061804 PMC5219805

[107] Dostal A Lacroix C Bircher L Pham VT Follador R Zimmermann MB . Iron modulates butyrate production by a child gut microbiota *in vitro*. mBio. (2015) 6:e01453–01415. 10.1128/mBio.01453-15 26578675 PMC4659462

[108] Dostal A Fehlbaum S Chassard C Zimmermann MB Lacroix C. Low iron availability in continuous in vitro colonic fermentations induces strong dysbiosis of the child gut microbial consortium and a decrease in main metabolites. FEMS Microbiol Ecol. (2013) 83:161–75. 10.1111/j.1574-6941.2012.01461.x 22845175 PMC3511601

[109] Rivera-Chávez F Zhang LF Faber F Lopez CA Byndloss MX Olsan EE . Depletion of butyrate-producing clostridia from the gut microbiota drives an aerobic luminal expansion of salmonella. Cell Host Microbe. (2016) 19:443–54. 10.1016/j.chom.2016.03.004 27078066 PMC4832419

[110] Bridgman SL Azad MB Field CJ Haqq AM Becker AB Mandhane PJ . Fecal short-chain fatty acid variations by breastfeeding status in infants at 4 months: differences in relative versus absolute concentrations. Front Nutr. (2017) 4:11. 10.3389/fnut.2017.00011 28443284 PMC5385454

[111] Garrido D Dallas DC Mills DA. Consumption of human milk glycoconjugates by infant-associated bifidobacteria: mechanisms and implications. Microbiology. (2013) 159:649–64. 10.1099/mic.0.064113-0 23460033 PMC4083661

[112] Newburg DS Morelli L. Human milk and infant intestinal mucosal glycans guide succession of the neonatal intestinal microbiota. Pediatr Res. (2015) 77:115–20. 10.1038/pr.2014.178 25356747

[113] Kozak K Charbonneau D Sanozky-Dawes R Klaenhammer T. Characterization of bacterial isolates from the microbiota of mothers' breast milk and their infants. Gut Microbes. (2015) 6:341–51. 10.1080/19490976.2015.1103425 26727418 PMC4826109

[114] Wang M Li M Wu S Lebrilla CB Chapkin RS Ivanov I . Fecal microbiota composition of breast-fed infants is correlated with human milk oligosaccharides consumed. J Pediatr Gastroenterol Nutr. (2015) 60:825–33. 10.1097/MPG.0000000000000752 25651488 PMC4441539

[115] Bashiardes S Thaiss CA Elinav E. It's in the milk: feeding the microbiome to promote infant growth. Cell Metab. (2016) 23:393–4. 10.1016/j.cmet.2016.02.015 26959178

[116] Cabrera-Rubio R Collado MC Laitinen K Salminen S Isolauri E Mira A. The human milk microbiome changes over lactation and is shaped by maternal weight and mode of delivery. Am J Clin Nutr. (2012) 96:544–51. 10.3945/ajcn.112.037382 22836031

[117] González R Mandomando I Fumadó V Sacoor C Macete E Alonso PL . Breast milk and gut microbiota in african mothers and infants from an area of high HIV prevalence. PLoS ONE. (2013) 8:e80299. 10.1371/journal.pone.0080299 24303004 PMC3841168

[118] Davis JCC Totten SM Huang JO Nagshbandi S Kirmiz N Garrido DA . Identification of oligosaccharides in feces of breast-fed infants and their correlation with the gut microbial community. Mol Cell Proteomics. (2016) 15:2987–3002. 10.1074/mcp.M116.060665 27435585 PMC5013312

[119] Milani C Duranti S Bottacini F Casey E Turroni F Mahony J . The first microbial colonizers of the human gut: composition, activities, and health implications of the infant gut microbiota. Microbiol Mol Biol Rev. (2017) 81:e00036–17. 10.1128/MMBR.00036-17 PMC5706746 29118049

[120] Garrido D Ruiz-Moyano S Lemay DG Sela DA German JB Mills DA. Comparative transcriptomics reveals key differences in the response to milk oligosaccharides of infant gut-associated bifidobacteria. Sci Rep. (2015) 5:13517. 10.1038/srep13517 26337101 PMC4559671

[121] Harmsen HJ Wildeboer-Veloo AC Raangs GC Wagendorp AA Klijn N Bindels JG . Analysis of intestinal flora development in breast-fed and formula-fed infants by using molecular identification and detection methods. J Pediatr Gastroenterol Nutr. (2000) 30:61–7. 10.1097/00005176-200001000-00019 10630441

[122] Fukuda S Toh H Hase K Oshima K Nakanishi Y Yoshimura K . Bifidobacteria can protect from enteropathogenic infection through production of acetate. Nature. (2011) 469:543–7. 10.1038/nature09646 21270894

[123] Matsuki T Yahagi K Mori H Matsumoto H Hara T Tajima S . A key genetic factor for fucosyllactose utilization affects infant gut microbiota development. Nat Commun. (2016) 7:11939. 10.1038/ncomms11939 27340092 PMC4931012

[124] Martin R Makino H Cetinyurek Yavuz A Ben-Amor K Roelofs M Ishikawa E . Early-life events, including mode of delivery and type of feeding, siblings and gender, shape the developing gut microbiota. PLoS ONE. (2016) 11:e0158498. 10.1371/journal.pone.0158498 27362264 PMC4928817

[125] Kumari M Kozyrskyj AL. Gut microbial metabolism defines host metabolism: an emerging perspective in obesity and allergic inflammation. Obes Rev. (2017) 18:18–31. 10.1111/obr.12484 27862824

[126] Thorburn AN McKenzie CI Shen S Stanley D Macia L Mason LJ . Evidence that asthma is a developmental origin disease influenced by maternal diet and bacterial metabolites. Nat Commun. (2015) 6:7320. 10.1038/ncomms8320 26102221

[127] Arrieta MC Stiemsma LT Dimitriu PA Thorson L Russell S Yurist-Doutsch S . Early infancy microbial and metabolic alterations affect risk of childhood asthma. Sci Transl Med. (2015) 7:307ra152. 10.1126/scitranslmed.aab2271 26424567

[128] Shah R Sabir S Alhawaj AF. Physiology, Breast Milk. Treasure Island, FL: StatPearls Publishing (2022). Available online at: http://www.ncbi.nlm.nih.gov/books/NBK539790/ (accessed June 18, 2022). 30969612

[129] Duale A Singh P Al Khodor S. Breast milk: a meal worth having. Front Nutr. (2022) 8:800927. 10.3389/fnut.2021.800927 35155521 PMC8826470

[130] Cacho NT Lawrence RM. Innate immunity and breast milk. Front Immunol. (2017) 8:584. 10.3389/fimmu.2017.00584 28611768 PMC5447027

[131] Gila-Diaz A Arribas SM Algara A Martín-Cabrejas MA López de Pablo ÁL Sáenz de Pipaón M . A review of bioactive factors in human breastmilk: a focus on prematurity. Nutrients. (2019) 11:1307. 10.3390/nu11061307 PMC6628333 31185620

[132] van Sadelhoff JHJ Wiertsema SP Garssen J Hogenkamp A. Free amino acids in human milk: a potential role for glutamine and glutamate in the protection against neonatal allergies and infections. Front Immunol. (2020) 11:1007. 10.3389/fimmu.2020.01007 32547547 PMC7270293

[133] Kaczmarczyk M Löber U Adamek K Wegrzyn D Skonieczna-Zydecka K Malinowski D . The gut microbiota is associated with the small intestinal paracellular permeability and the development of the immune system in healthy children during the first two years of life. J Transl Med. (2021) 19:177. 10.1186/s12967-021-02839-w 33910577 PMC8082808

[134] Martin FPJ Moco S Montoliu I Collino S Da Silva L Rezzi S . Impact of breast-feeding and high- and low-protein formula on the metabolism and growth of infants from overweight and obese mothers. Pediatr Res. (2014) 75:535–43. 10.1038/pr.2013.250 24375085

[135] Chow J Panasevich MR Alexander D Vester Boler BM Rossoni Serao MC Faber TA . Fecal metabolomics of healthy breast-fed versus formula-fed infants before and during in vitro batch culture fermentation. J Proteome Res. (2014) 13:2534–42. 10.1021/pr500011w 24628373

[136] Macé K Steenhout P Klassen P Donnet A. Protein quality and quantity in cow's milk-based formula for healthy term infants: past, present and future. Nestle Nutr Workshop Ser Pediatr Program. (2006) 58:189–203. 10.1159/000095063 16902335

[137] Payne AN Chassard C Zimmermann M Müller P Stinca S Lacroix C. The metabolic activity of gut microbiota in obese children is increased compared with normal-weight children and exhibits more exhaustive substrate utilization. Nutr Diabetes. (2011) 1:e12. 10.1038/nutd.2011.8 23154580 PMC3302137

[138] Fernandes J Su W Rahat-Rozenbloom S Wolever TMS Comelli EM. Adiposity, gut microbiota and faecal short chain fatty acids are linked in adult humans. Nutr Diabetes. (2014) 4:e121. 10.1038/nutd.2014.23 24979150 PMC4079931

[139] Rahat-Rozenbloom S Fernandes J Gloor GB Wolever TMS. Evidence for greater production of colonic short-chain fatty acids in overweight than lean humans. Int J Obes. (2014) 38:1525–31. 10.1038/ijo.2014.46 PMC3970979 24642959

[140] Schwiertz A Taras D Schäfer K Beijer S Bos NA Donus C . Microbiota and SCFA in lean and overweight healthy subjects. Obesity. (2010) 18:190–5. 10.1038/oby.2009.167 19498350

[141] Teixeira TFS Grześkowiak Ł Franceschini SCC Bressan J Ferreira CLLF Peluzio MCG. Higher level of faecal SCFA in women correlates with metabolic syndrome risk factors. Br J Nutr. (2013) 109:914–9. 10.1017/S0007114512002723 23200109

[142] Vogt JA Wolever TMS. Fecal acetate is inversely related to acetate absorption from the human rectum and distal colon. J Nutr. (2003) 133:3145–8. 10.1093/jn/133.10.3145 14519799

